# Expression Dynamics and Genetic Compensation of Cell Cycle Paralogues in *Saccharomyces cerevisiae*

**DOI:** 10.3390/cells14060412

**Published:** 2025-03-11

**Authors:** Gabriele Schreiber, Facundo Rueda, Florian Renner, Asya Fatima Polat, Philipp Lorenz, Edda Klipp

**Affiliations:** Theoretical Biophysics, Humboldt-Universität zu Berlin, Invalidenstr. 42, 10115 Berlin, Germany; gabriele.schreiber@hu-berlin.de (G.S.); facundo.rueda@mdc-berlin.de (F.R.); florian.renner.1@hu-berlin.de (F.R.); polatasy@hu-berlin.de (A.F.P.); philipp.lorenz@lorenz-berlin.eu (P.L.)

**Keywords:** *Saccharomyces cerevisiae*, paralogous genes, cell cycle, cyclins, single molecule inexpensive fluorescent in situ hybridization (smiFISH), *CLB1/CLB2*, *CLB3/CLB4*, *CLB5/CLB6*, *CLN1/CLN1*, *SWI5/ACE2*

## Abstract

Cell cycle progression of the yeast *Saccharomyces cerevisiae* is largely driven by the expression of cyclins, which in turn bind the cyclin-dependent kinase CDK1 providing specificity. Due to the duplication of the yeast genome during evolution, most of the cyclins are present as a pair of paralogues, which are considered to have similar functions and periods of expression. Here, we use single molecule inexpensive fluorescence in situ hybridization (smiFISH) to measure the expression of five pairs of paralogous genes relevant for cell cycle progression (*CLN1*/*CLN2*, *CLB5*/*CLB6*, *CLB3*/*CLB4*, *CLB1*/*CLB2* and *ACE2*/*SWI5*) in a large number of unsynchronized single cells representing all cell cycle phases. We systematically compare their expression patterns and strengths. In addition, we also analyze the effect of the knockout of one part of each pair on the expression of the other gene. In order to classify cells into specific cell cycle phases, we developed a convolutional neural network (CNN). We find that the expression levels of some cell-cycle related paralogues differ in their correlation, with *CLN1* and *CLN2* showing strong correlation and *CLB3* and *CLB4* showing weakest correlation. The temporal profiles of some pairs also differ. Upon deletion of their paralogue, *CLB1* and *CLB2* seem to compensate for the expression of the other gene, while this was not observed for *ACE2*/*SWI5*. Interestingly, *CLB1* and *CLB2* also seem to share work between mother and bud in the G2 phase, where *CLB2* is primarily expressed in the bud and *CLB1* in the mother. Taken together, our results suggest that paralogues related to yeast cell cycle progression should not be considered as the same but differ both in their expression strength and timing as well in their precise role in cell cycle regulation.

## 1. Introduction

About 100 million years ago a whole genome duplication (WGD) in an ancestral yeast occurred, giving rise to *Saccharomyces*. The mechanism behind this genome duplication was long under debate, with theories suggesting a mating event between closely related *Klyveromyces* strains followed by genomic reorganization or independent local duplications [1,2]. More recent studies, based on the availability of full genome sequences and a search for signs of ancient gene duplications, favor the hypothesis of a duplication of the full genome followed by a massive loss of about 90% of duplicated genes [3]. About 13% of yeast proteins are encoded by paralogous genes, including pairs of transcription factors, protein kinases, myosins, cyclins and pheromones [4].

Gene duplication generally is seen as an important source of evolutionary novelty. The idea is that after duplication one gene copy is free to diverge either to specify on only a part of the original functions (subfunctionalization), develop new functions (neofunctionalization) or gain positive effects by using pre-existing functions in a new context (exaptation). In theory, gene duplication and the persistence of the duplicated genes is related to functional innovations, otherwise the gene duplication is considered to be only a burden without benefit. Nevertheless, for about 70% of the 457 duplicated gene pairs, no strong divergence from the proposed ancestor *Klyveromyces waltii* was found [3]. In these cases, divergence may occur in regulatory regions or the duplication may simply increase gene dosage. The ability of *Saccharomyces cerevisiae* to perform anaerobic fermentation, a biochemical pathway central to brewing and biofuel production, is believed to have originated as an adaptation following WGD [5].

**Figure 1:**
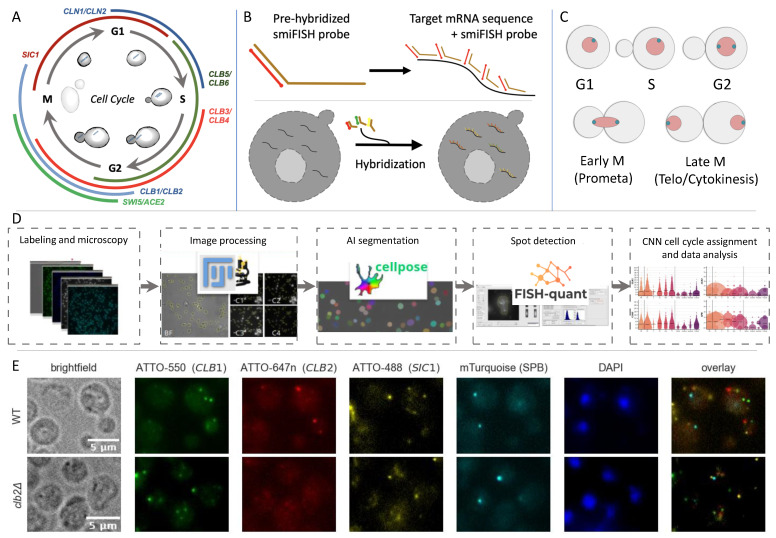
Project Overview. (**A**) Yeast cell cycle schematic with oscillating expression of pairs of paralogous cyclins, the inhibitor *SIC1* and the paralogous transcription factor pair *SWI5* and *ACE2*. (**B**) smiFISH Principle: 24 unlabeled DNA probes, containing a complementary part to the target mRNA and an extension complementary to a fluorescent labeled DNA, are pre-hybridized, before using them for FISH labeling of yeast spheroplasts. (**C**) Schematic representation of the classification criteria used to assign individual cells to specific cell-cycle phases for CNN training. (**D**) Experimental and analysis pipepline. (**E**) Example fluorescence microscopy images showing mRNA localization in WT and *clb2*Δ strains. Columns represent different imaging channels: brightfield, ATTO-550 (*CLB1*), ATTO-647n (*CLB2*), ATTO-488 (*SIC1*), mTurquoise (SPB), DAPI, and an overlay of all channels. Scale bar: 5 μm.

In our study, we focus on cell cycle regulation, and investigate five pairs of paralogous genes involved in this process: *CLN1/CLN2*, *CLB1/CLB2*, *CLB3/CLB4*, *CLB5/CLB6*, as well as *ACE2/SWI5* (Figure 1). While these paralogous pairs are typically considered in conceptual and computational models [6,7,8] as a single entity, they may have distinct roles. The B-type cyclins *CLB1* and *CLB2* regulate the transition from the G2 phase to mitosis. However, *CLB2* mRNA is enriched in the bud, where it could function as a sensor for bud size and readiness for the M-phase transition, while *CLB1* lacks this enrichment [9]. This suggests that these two cyclins may have complementary but non-identical roles in mitotic regulation. Similarly, *CLN1* and *CLN2*, although highly similar, show functional differences, particularly with regard to subcellular localization and their roles in promoting cell cycle progression [9,10]. The paralogues *ACE2* and *SWI5* are expressed at the M/G1 phase boundary with partly different roles. The transcription factor Ace2p is required for septum destruction after cytokinesis and it regulates transcription of genes involved in polarity and morphogenesis, while Swi5p activates transcription of genes required for mating type switching (HO endonuclease gene) and the mitotic exit by activation of *SIC1* transcription [11]. There are some paralogue pairs where evolutionary divergence from the ancestor is happening only in one gene, while the other one stays mostly unaltered. For the majority of paralogues and also for our targets in this study this is not the case. The paralogue pair *CLB1/CLB2*, for example, has protein identity to the *K. waltii* ancestor of 59% and 63%, respectively. The lowest identity to the ancestor for the targets in our study was found for the transcription factors *ACE2/SWI5*, with protein identities of 35% and 31%, respectively [3]. The protein identity between *ACE2* and *SWI5* is the lowest in our target set at 46.84%, though not significantly lower than that of the cyclins. Interestingly coding sequence identity between *ACE2* and *SWI5* is with 70.12% much higher. Information about target genes and identity between paralogues is displayed in Table 1.

Genetic robustness allows cells to maintain functionality despite perturbations and gene duplication plays a major role in this process. If one paralogue is deleted or inactivated, the other may compensate by increasing its expression, a mechanism known as genetic compensation. However, the frequency of genetic compensation and the underlying molecular mechanisms are still not fully understood [12]. Earlier studies suggested that genetic compensation has only a minor role in genetic robustness [13], but other findings challenge this view. Kafri et al. [14] systematically analyzed paralogous compensation in yeast and found that it occurs mainly between paralogues with different expression profiles, pointing to regulatory reprogramming. More recently, Vande Zande et al. [15] showed that the yeast paralogue *TDH2* is actively upregulated when *TDH3* is deleted, mediated by transcriptional regulators. DeLuna et al. [16] showed that in 10% of cases, this increased transcript level translates into higher protein abundance, which is essential for functional compensation.

In this study, we used single-molecule inexpensive fluorescence in situ hybridization (smiFISH) [17] to investigate the expression profiles of *CLB1/CLB2*, *CLB3/CLB4*, *CLB5/CLB6*, *CLN1/CLN2* and *SWI5/ACE2* (Figure 1). According to Tsanov et al., it holds that “because of the low cost of the unlabelled primary probes, more probes per gene can be used, thus resulting in a substantial increase in signal quality”. Figure 1A gives a schematic view on yeast cell cycle and Figure 1B introduces the smiFISH principle. To explore the role of genetic compensation, we used deletion strains of the respective paralogues and analyzed their expression patterns. Additionally, we developed a convolutional neural network (CNN) to classify cells into specific cell cycle phases, with the classification criteria symbolized in Figure 1C. The full series of steps taken is shown in Figure 1D. We found distinct expression dynamics across paralogues, with differences in their regulation and cell cycle timing. Notably, we observed genetic compensation in some paralogues upon gene deletion, while others show little to no compensatory response. Figure 1E shows an example of images employed for the analysis both for WT cells and for *clb2*Δ strains.

## 2. Materials and Methods

### 2.1. Yeast Strain

We used the fully sequenced haploid yeast strain BY4741 (MAT a; *his3*Δ*1*; *leu2*Δ*0*; *met15*Δ*0*; *ura3*Δ*0*) and its derivatives for our study. Deletion strains with the same genetic background were obtained from EUROSCARF (http://www.euroscarf.de, accessed on 5 March 2025). To visualize spindle-pole bodies and as a marker for cell-cycle phase assignment, we tagged Spc42 with mTurquoise (Spc42::Spc42-mTurquiose KanMX4) [18] (Figure 1E, Supplementary Methods).

### 2.2. Cell Growth and Preparation of Yeast Cells for smiFISH Labeling

Cell growth, preparation and hybridization was essentially performed as described by Trcek et al. [19], with small modifications. Shortly, yeast cells were grown in YPD medium at 30 °C under shaking with 220 rpm in erlenmeyer flasks until $OD_{600}$ < 0.8. The cells in exponential growth phase were fixated with 4% Paraformaldehyde ((Electron Microscopy Sciences, Hatfield, PA, USA, cat. no. 15714) at room temperature for 45 min under constant rotation. Afterwards cells were harvested and washed as described by Trcek et al. [19]. Cell wall digestion was performed for 9 min with 3000 U/mL lyticase (Merck KGaA; Sigma-Aldrich, Darmstadt, Germany, cat. no. L2524) in spheroplasting buffer [19] at 30 °C with constant soft rotation. After spheroplasting, cells were washed and finally resolved in 70% ethanol. Spheroplasted cells were stored for several weeks at −20 °C.

### 2.3. smiFISH Probe Preparation

We used smiFISH probes (single molecule inexpensive Fluorescence in situ hybridization) published by Tsanov et al. in our study [17]. For each target mRNA 24 DNA probes were generated with an R-script *Oligostan* which was supplied by the authors [17]. With the R-script the so called FLAP sequence, i.e., a short shared sequence, was added to target specific oligonucleotides (FLAP-X, FLAP-Y or FLAP-Z). The 24 unlabeled DNA probes with the FLAP overhangs were purchases as DNA oligonucleotids from Thermo Fisher (Invitrogen; Thermo Fisher Scientific Inc., Waltham, MA, USA), dissolved and mixed as described by Tsanov et al. [17]. FLAPs conjugated to fluorescent dyes were purchased from Biomers (https://www.biomers.net, accessed on 5 March 2025; biomers.net GmbH, Ulm, Germany). We used FLAP oligonucleotides labeled with ATTO-550, ATTO-647n and ATTO-488 in order to image three different targets in one FISH experiment. We prepared all possible FLAP/dye combinations. Hybridization of unlabeled probes with the ATTO labeled FLAPs was performed as described [17] with a final DNA concentration of 40 pmol per 10 µL.

### 2.4. smiFISH Hybridization

For FISH labeling of specific mRNA the frozen spheroplasts were rehydrated in 1 mL 2× Saline-sodium citrate (SSC, Carl Roth GmbH + Co. KG, Karlsruhe, Germany cat. no. 1054.1) for about 2 min in 1.5 mL tubes. Afterwards cells were gentle centrifuged for 4 min at 1500× *g* at 4 °C. Buffer was removed completely and 50 µL hybridization solution [19] with 0.5 µL of each fluorescent labeled smiFISH probe mix was added. We used three different fluorescent dyes for labeling of three targets at once. FISH hybridization was incubated for >4 h or over night at 37 °C on a gently rotating platform. After labeling, cells were washed 3 times 30 min at 37 °C under gentle rotation with washing buffer containing 1× SSC and 10% formamide. After washing with formamide containing buffers, cells were washed and rehydrated twice in 2× SSC buffer. Centrifugation in all steps was performed for 4 min at 1500× *g* at room temperature. After the last washing step, buffer volume was reduced to 150 µL. Cells were resuspended carefully in the remaining buffer and aggregates were removed by sonification for 2 min with 10% power. Cells were pipetted on (3-Aminopropyl) triethoxysilane (APTES, Merck KGaA; Sigma-Aldrich, Darmstadt, Germany, cat. no. 440140) activated coverslips with silicon chamber (Figure S1). Fixation on the activated surface was performed over night at 4 °C protected from light.

### 2.5. Imaging

For imaging of the smiFISH labeled yeast cells, we used APTES activated coverslips [20] with a silicon chamber (Invitrogen; Thermo Fisher Scientific Inc., Waltham, MA, USA, cat. no. P18179) glued on, to build a chamber for the cell solution (Figure S1 and Supplementary Methods) Binding of the cells was performed over night at 4 °C. Before imaging, unbound cells were removed by rinsing the chamber with 2× SSC. 4′,6-Diamidine-2′-phenylindole dihydrochloride (DAPI, Sigma cat. no. 32670) labeling was performed directly before imaging. DAPI stock solution (300 µM) was diluted 1:1000 and added to the chamber. After 4 min incubation DAPI solution was removed and the cells were washed again with 2× SSC. Imaging with an Olympus IX-83 microscope (Olympus; Evident Corporation, Tokyo, Japan) was performed right after DAPI labeling. We use an inverted microscope equipped with an 100× UPLSAPO objective (NA 1.4) an EMCCD camera (Andor iXon Ultra 888, Oxford Instruments Group; Andor, Abingdon, UK) a motorized stage (Märzhäuser TANGO, Märzhäuser Wetzlar GmbH & Co. KG, Wetzlar, Germany), a LED light machine (Lumencor SolaFISH, Lumencor, Beaverton, OR, USA), Olympus filter cubes and an additional filter wheel (Prior Scientific Instruments GmbH, Jena, Germany). For automatic acquisition of manually chosen positions we use a predefined protocol in cellSens software (Olympus; Evident Corporation, Tokyo, Japan). For each positions and each channel a stack with 32 layers with 0.25 µm distance was imaged: BF (bright field), Cy3 (Atto550), Cy5 (Atto647n), YFP (Atto488), CFP (mTurquoise), DAPI (DAPI). For the first four fluorescent channels, Olympus filter cubes were used, for the DAPI channel we used the additional filter wheel.

### 2.6. Image Processing

We use FIJI in combination with own python scripts for image processing [21]. From the Olympus .vsi format, we separate channels and save the stacks as .tif files. The brightfield stack, the CFP stack with spindle pole bodies and the DAPI stack were combined and maximum projected for segmentation with Cellpose 3.0. Cellpose 3.0 [22] is an AI segmentation model with a selection of pretrained models (model zoo) for different cell types. We trained the “yeast_BF_cp3” model with our images quite extensive until we got satisfying results. Cellpose outputs can be saved as mask images and as text files. The mask images are imported into FISH-quant [17] segmentation tool to get outline files, which can be used for FISH-quant spot detection. Since FISH-quant is using wavelength dependent theoretical point-spread functions, we duplicated the outline files for all channels to be analyzed with the excitation and emission maxima of the dyes used as meta data in the header. FISH-quant detection was performed according to the advices given in the manual, several cells with clear spots were chosen and the threshold was set manually to meet the requirements. Threshold determination was done for each channel and each experiment newly. After processing of all channels, output tables were combined, and all results were plotted.

### 2.7. Data Analysis

We used Seaborn boxenplots to visualize the distribution of the data. A boxenplot is similar to a standard boxplot, but it divides the data into a greater number of quantiles, providing more detailed information about the tails of the distribution. This method is especially useful for visualizing datasets with outliers or extreme values, as it offers a clearer representation of the data’s structure in both the central and extreme portions of the distribution. Besides the spot numbers per cell and channel, we recorded segmented cell area, spot intensity and correlation of the three target genes in the same experiment.

To assess the co-expression of paralogue pairs, we calculated the Pearson correlation coefficient using the *pearsonr* function from the *scipy.stats* library in Python. This method quantifies the linear relationship between the expression levels of two genes across individual cells. Specifically, we computed correlation coefficients based on the actual transcript counts detected for both paralogues. The resulting values range from −1 (strong negative correlation) to +1 (strong positive correlation), with values near 0 indicating little to no correlation. Additionally, *pearsonr* provides a *p*-value to assess the statistical significance of each correlation.

To compare mRNA expression levels between groups, we applied the Mann–Whitney U test, a nonparametric statistical method that evaluates whether one group tends to have higher values than the other without assuming a normal distribution of the data. The test ranks all observations from both groups together and then compares the sum of these ranks. The test is done under the null hypothesis that both groups are expected to have similar rank distributions, whereas a significant deviation suggests a systematic difference in expression levels. A *p*-value below 0.05 was considered statistically significant, indicating a meaningful difference in mRNA counts between the groups.

Given the large number of single-cell measurements in our dataset, statistical significance was consistently high, with *p*-values frequently falling well below conventional thresholds. Therefore, rather than interpreting significance in absolute terms, we focused on the relative magnitude of expression differences between gene pairs.

### 2.8. Convolutional Neural Networks (CNN) for Cell Cycle Stage Assignment

#### 2.8.1. Data Preprocessing

To prepare the microscopy images of yeast cells for classification, we utilized a series of preprocessing steps. Each cell segmented by Cellpose 3.0 [22] was cropped based on its segmented boundary, resulting in image frames that served as inputs to the CNN. To reduce dimensionality and computational complexity, we applied a standard deviation projection along the *z*-axis to single-cell image stacks, converting them into 2D images. Local thresholding was applied to the spindle pole body images to highlight spots with high contrast in respect to their environment. To emphasize the cell of interest, the segmented cell border was overlaid onto each projected image. Three of the imaging channels, bright field (BF), spindle pole body (SPB), and DAPI, were stacked to create a composite input image for the CNN with dimensions 80 × 80 × 3.

#### 2.8.2. Ground Truth

Each segmented cell was manually inspected and assigned a ground truth label corresponding to one of the following cell cycle phases: G1, S, G2, Early-M, Late-M, S-Bud, G2-Bud, Early-M-Bud, or Late-M-Bud. The classification scheme is illustrated in Figure 1C. To address class imbalance, additional screening efforts targeted underrepresented cell cycle phases to enhance training robustness. The final dataset comprises approximately 3000 annotated cells.

#### 2.8.3. CNN Architecture

The CNN was designed to classify yeast cells into their respective cell cycle phases. The architecture begins with 5 convolutional blocks, each consisting of:

- A convolutional layer,
- batch normalization,
- attention modules (spatial attention and channel attention),
- ReLU activation function and
- a pooling layer.

The first convolutional layer contains 32 filters, and the number of filters doubles with each subsequent convolutional block. Following the convolutional blocks, the network includes one fully connected block, comprising a forward fully connected layer, a ReLU activation function, and a dropout layer. The network concludes with a fully connected output layer, where the number of nodes corresponds to the number of cell cycle phases to predict.

#### 2.8.4. CNN Training Workflow

To address class imbalance, batches were constructed to include an equal representation of each cell phase. Before being passed to the model, images underwent data augmentation to enhance generalization. Augmentation strategies included random rotations, vertical and horizontal flips and adjustments to saturation and contrast. The model was trained using the Adam optimizer, with focal loss as the loss function to address class imbalance and emphasize hard-to-classify examples.

#### 2.8.5. CNN Performance

**Figure 2:**
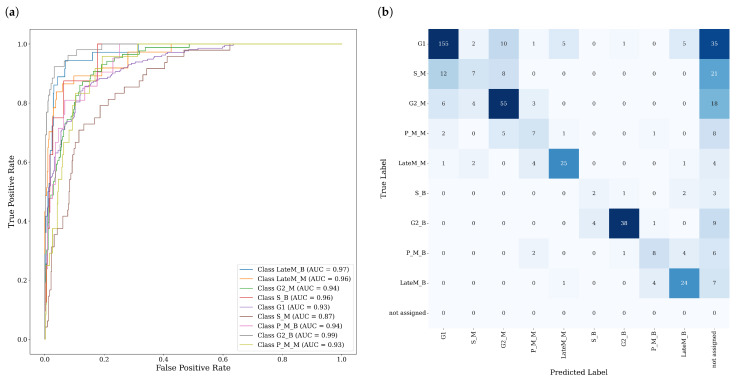
(**a**) Receiver Operating Characteristic (ROC) curves for each cell-cycle phase, computed using a one-vs-all approach. The legend displays the corresponding Area Under the Curve (AUC) scores. (**b**) Confusion matrix illustrating the manually assigned cell-cycle phases versus CNN predictions for the validation set. True positives are located along the diagonal.

The performance of the CNN model was evaluated on independent validation sets. To ensure robust benchmarking, a 5-fold cross-validation approach was employed, and the validation metrics were averaged across folds. The model achieved an overall mean accuracy of 64% and a mean F1 score of 65%. For the final model that we employed, we implemented a class specific thresholding, filtering out 20% of the cells with the lowest confidence in their prediction for each cell-cycle phase. After filtering, an accuracy of 77%, a F1 score of 77% and a Area under the Curve (AUC) score of 95% was achieved. However, performance varies significantly across different cell cycle phases. Higher performance is observed for G1 and mitotic cells, whereas lower performance is noted for S-phase mother and bud cells, reflecting the greater complexity of these classes. A detailed breakdown of performance by cell-cycle phase is presented in Figure 2. The full implementation of the CNN can be reviewed at: https://ford.biologie.hu-berlin.de/frenner/yeastparalogues25 (accessed on 13 February 2025).

## 3. Results

To investigate differences in the mRNA expression of key cell cycle regulators, we measured absolute transcript numbers for *CLB1*, *CLB2*, *CLB3*, *CLB4*, *CLB5*, *CLB6*, *CLN1*, *CLN2*, *ACE2*, *SWI5* and *SIC1* using smiFISH.

### 3.1. Distinct mRNA Expression Patterns of Cell Cycle Regulators

**Figure 3:**
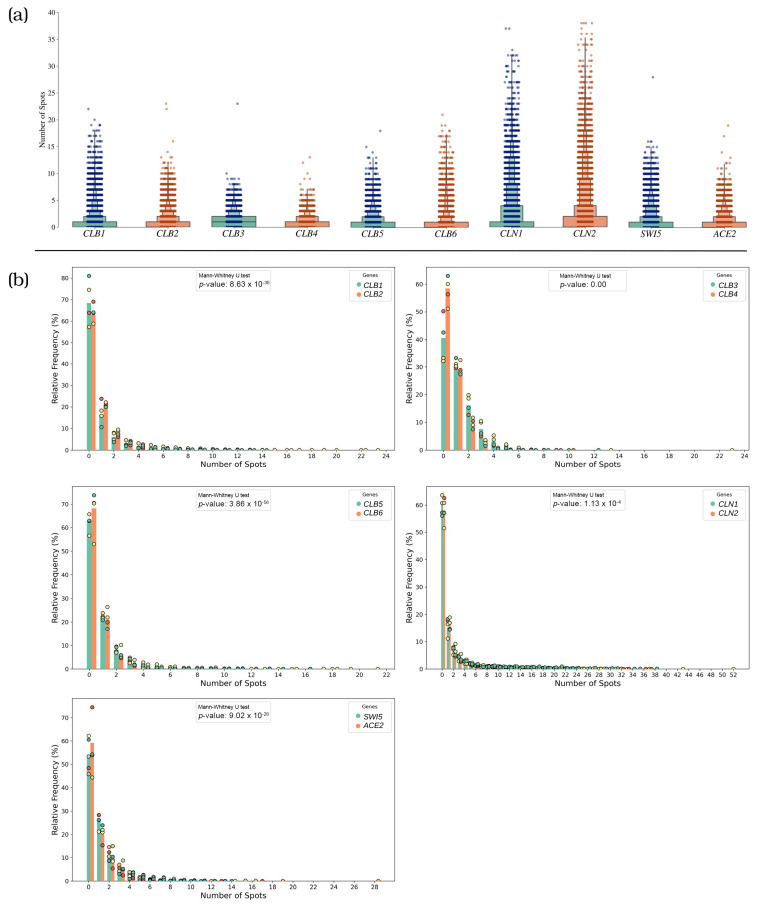
Expression patterns of mRNA in paralogous gene pairs analyzed in this study. (**a**) Boxenplots with number of spots observed for each gene. (**b**) Histograms with relative frequency of number of spots observed. Dots indicate individual experiments. The *p*-values of Mann-Whitney U test indicate for each pair significant differences of both smiFISH data series, but to the least degree for genes *CLN1/CLN2*.

Analysis of mRNA distributions revealed varied expression patterns across the genes studied (Figure 3). The boxenplots illustrate mRNA detection across the entire cell population coming from three to five replicate experiments, highlighting the variability in expression levels. Results from the individual experiments can be found in Figure S2. Notably, *CLN1* and *CLN2* showed the highest overall expression, with mean values of 1.68 and 1.89 spots per cell, respectively. In contrast, *CLB4* emerged as the least detected gene, with a mean value of 0.59 spots per cell. This variation highlights the diverse expression patterns among the genes. It had been suggested that gene expression can occur in two primary modes: continuous (constitutive) expression, where transcripts accumulate steadily over time, or bursty expression, where transcription happens in short, intense pulses [23]. Most of the regulators exhibited intense transcriptional bursts with some cells containing up to 52 transcripts for *CLN2*, while *CLB3* and *CLB4* displayed lower overall expression with less pronounced peaks of about 10 transcripts, suggesting an expression pattern with less intense bursts, closer to a constitutive rather than bursty expression. The histograms further elucidate the distribution of mRNA levels among individual cells. *CLB1* and *CLB2*, *CLB4*, *CLB5*, *CLB6*, were all detected in between 38% to 32% of the cells. Interestingly, the paralogue pair with the lowest expression levels (*CLB3/CLB4*) had the highest proportion of cells without detectable spots, with 60% and 42% of cells showing no signal, respectively. These findings could suggest the hypothesis of a more constitutive expression pattern, characterized by lower but more widely distributed detection of spots across the entire cell population, though the very low numbers of measured mRNA molecules don’t allow for strong conclusion. Furthermore, despite their significantly higher mean expression levels, *CLN1* and *CLN2* were only detected in approximately 40% of the cells. These findings suggest that while *CLN1* and *CLN2* exhibit intense transcriptional bursts in individual cells, their activity may be more phase specific.

### 3.2. Correlation Analysis Reveals Co-Expression or Distinct Regulation of Paralogous Gene Pairs

To explore relationships between the expression levels of paralogous genes, we calculated the mean single-cell Pearson correlation coefficient (Table 2). The values reveal that *CLB1/CLB2*, *CLB5/CLB6*, and *SWI5/ACE2* exhibit moderate positive correlations, with values around 0.5, suggesting co-regulation. The *CLN1/CLN2* pair showed the highest correlation coefficient, with a value of 0.68. In contrast, *CLB3/CLB4* displayed weak correlation, with a value of 0.26, highlighting distinct expression patterns. These findings suggest divergent co-expression patterns and underlying regulatory mechanisms for paralogue genes within the cell cycle.

In addition to examining correlations among paralogous genes, we investigated the relationship between each gene’s expression and the expression of *SIC1*, a key cell cycle regulator. The correlation values (Tables S1 and S2) revealed that all the genes included in this study showed minimal correlation with *SIC1*, reflecting either distinct regulatory mechanisms that may function independently of *SIC1* throughout the cell cycle or simply a clear temporal delay between their expression.

### 3.3. Phase-Specific Expression Patterns of Paralogous Genes Across the Cell Cycle

To examine gene expression throughout the cell cycle, we first combined smiFISH detection with spindle pole body (SPB) recognition, see Figure 1E. SPB visualization was achieved by tagging Spc42p with mTurquoise as explained in Materials and Methods and the Supplementary Materials. Cells with one SPB correspond mainly to G1, S, and M phases, cells with two SPBs only account for G2 phase, and cells with zero SPB are mostly S/Buds or G2/Buds [19]. The analysis (Figure S3) reveals distinct expression patterns for specific paralogues across the cell cycle. *CLB1/CLB2* are predominantly expressed in cells with two SPB, suggesting peak transcription during G2 phase. In contrast, the *CLN1/CLN2* pair shows higher mean values for cells with 1 SPB, suggesting stronger transcriptional activity during G1, S or M phases. These findings highlight phase-specific regulation of paralogous genes, with distinct transcriptional activity associated with different cell cycle stages.

**Figure 4:**
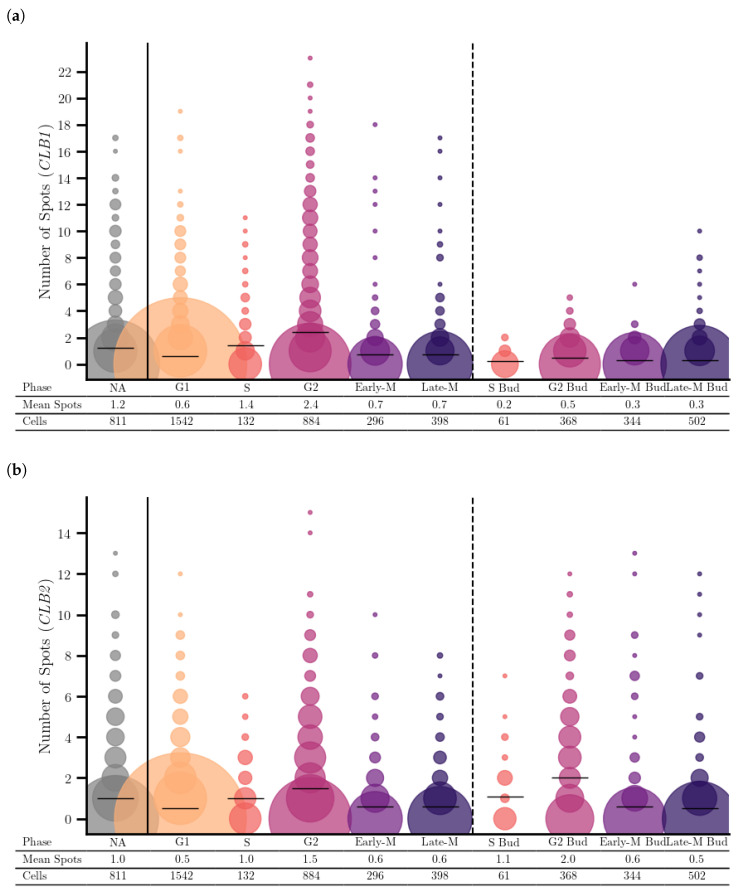
Distribution and mean number of mRNA spots per cell for (**a**) *CLB1* and (**b**) *CLB2* resolved over the cell-cycle phases.

To achieve a higher resolved assessment of cell-cycle stage specific expression we trained and employed a CNN for automatic cell-cycle assignment of cells. The resulting analysis supports the previous finding of phase specific regulation patterns, with higher expression for *CLB1* and *CLB2* in G2 mothers, while it also shows the distinct transcriptional activity in the G2 Buds (Figure 4). The cell-cycle specific mRNA expression of all analyzed genes can be found in Supplementary Figures S4 and S5. For most of the genes analyzed here, the expression pattern fitted quite well to previously published data [24,25]. In their studies, expression peaks in G1 phase were found for *CLB5/CLB6* and *CLN1/CLN2*, expression in S-G2 for *CLB3/CLB4*, expression in G2 for *CLB1/CLB2* and in G2-M *ACE2/SWI5*. For *CLB1/CLB2*, *CLB3/CLB4* and *ACE2/SWI5*, our data confirm the expression pattern published. For *CLB5/CLB6*, we see only very low transcript numbers and no clear peak phase as well as for *CLN1/CLN2* even though transcript numbers for the latter are higher.

### 3.4. Distinct Localization of *CLB1* and *CLB2*: *CLB2* Is Enriched in the Bud

The localization of *CLB1* and *CLB2* shows distinct patterns throughout the cell cycle. Both genes have expression peaks in G2 phase but their subcellular distributions are different. *CLB1* has a higher expression level than *CLB2* and is mainly localized to the mother cell, while *CLB2* is found in both the mother cell and the bud, suggesting a functional specialization, with *CLB2* likely involved in processes in the bud during the G2 phase.

**Figure 5:**
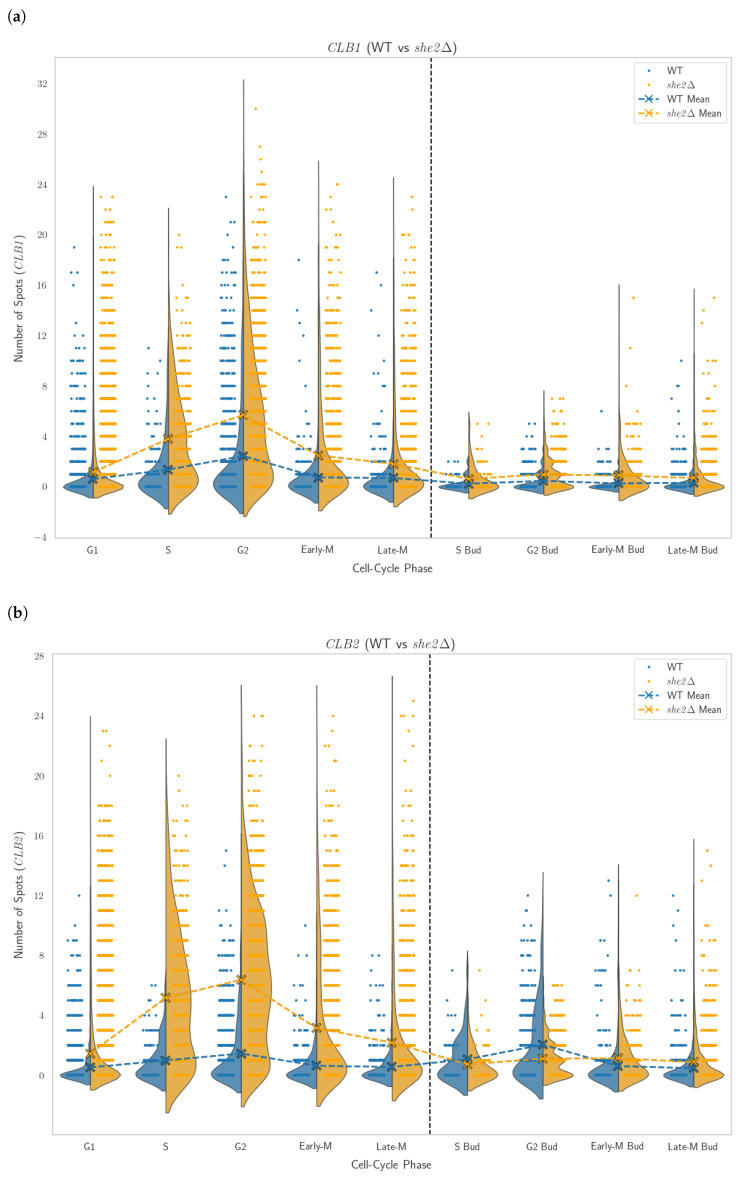
Distribution of mRNA spots counts across cell cycle phases for *CLB1* and *CLB2*. (**a**) *CLB1* expression in WT and *she2*Δ strains. (**b**) *CLB2* expression in WT and *she2*Δ strains.

She2p is a RNA-binding protein and part of She2p-She3p mRNA transport complex that brings certain proteins to the bud. She2p was shown to be involved in transport of *CLB2* mRNA to the bud [26] but not *CLB1*. Since we saw difference in compensation between the *clb1*Δ and the *clb2*Δ strain, we went on to check their expression pattern in the *she2*Δ strain (Figure 5). We see that *CLB1* is massively upregulated in the mother cell, but only slightly in the bud. *CLB2* in the mother is even more upregulated, with a few spots in the bud. We speculate that the upregulation in the mother leads to higher density leading to a diffusion of mRNAs into the bud, even without support from She2.

### 3.5. Genetic Compensation in Deletion Strains

**Figure 6:**
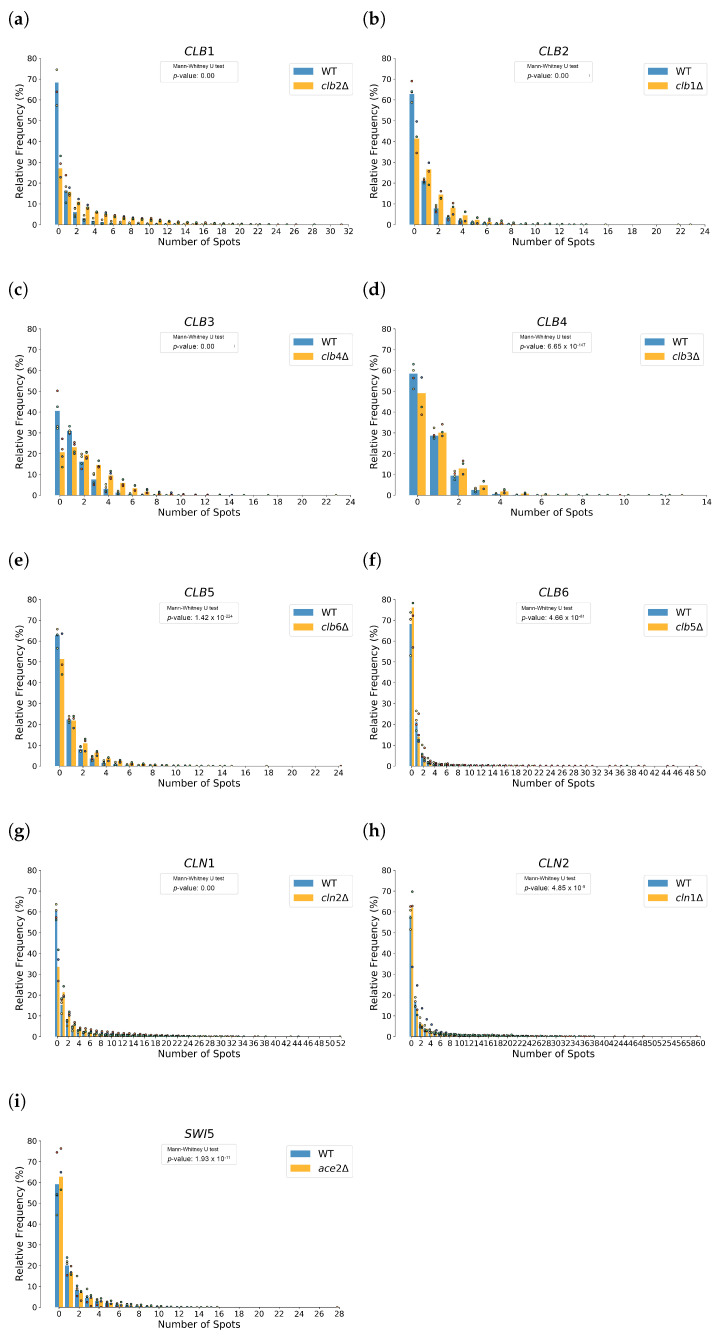
Distribution of mRNA spot counts for WT and deletion strains of paralogous genes with Mann-Whitney U-test. The x-axis represents the number of detected mRNA spots per cell, while the y-axis shows the relative frequency of cells with a given spot count. Each bar represents the pooled data from multiple experiments, with the dots indicating the mean value for one individual experiment. (**a**) Expression of *CLB1* in WT and *clb2*Δ strains. (**b**) Expression of *CLB2* in WT and *clb1*Δ strains. (**c**) Expression of *CLB3* in WT and *clb4*Δ strains. (**d**) Expression of *CLB4* in WT and *clb3*Δ strains. (**e**) Expression of *CLB5* in WT and *clb6*Δ strains. (**f**) Expression of CLB6 in WT and clb5Δ strains. (**g**) Expression of *CLN1* in WT and *cln2*Δ strains. (**h**) Expression of *CLN2* in WT and *cln1*Δ strains. (**i**) Expression of *SWI5* in WT and *ace2*Δ strains. The *p*-values of the Mann-Whitney U-test show strong differences between WT and deletion strain for *CLB1*, *CLB2*, *CLB3* with *p*-values near cero. For *CLB4*, *CLB5* and *CLB6* *p*-values are still very small with the least significance for CLB6 which still shows a *p*-value small enough to hold the assumption that the two distributions for WT and deletion strain differ from each other. For *CLN2*, and *SWI5* significance is smaller but still high enough for the assumption that mRNA distribution of the two strains differ from each other significantly.

**Figure 7:**
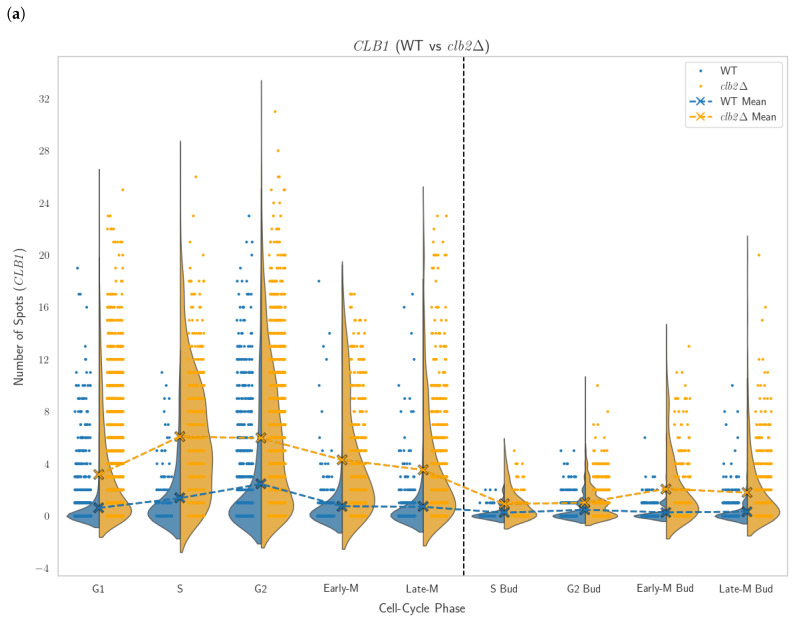

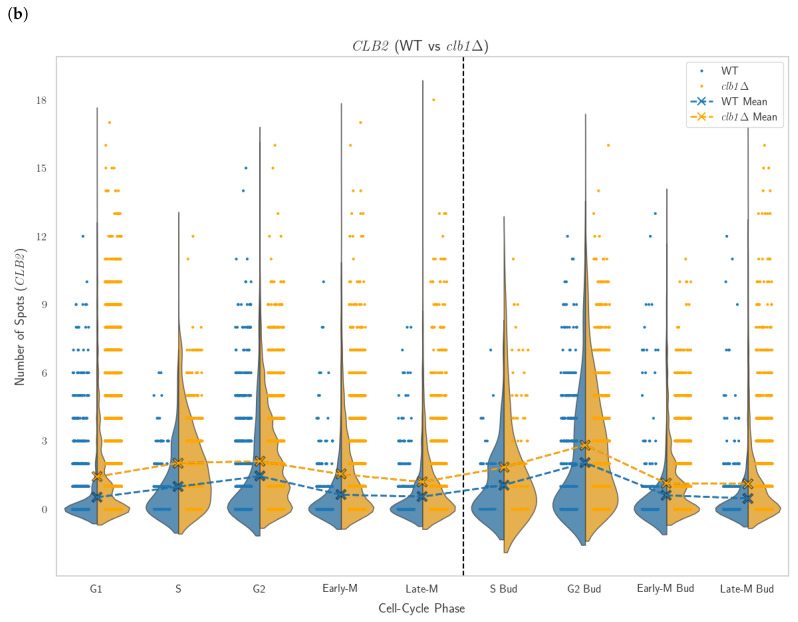
Distribution of mRNA spots counts across cell cycle phases for *CLB1* and *CLB2*. (**a**) *CLB1* expression in WT and *clb2*Δ strains. (**b**) *CLB2* expression in WT and *clb1*Δ strains.

To investigate the potential genetic compensation between paralogues, we compared mRNA expression levels between WT and deletion strains, where each gene was examined in the context of its paralogue’s deletion. The relative frequency of mRNA detections for each gene in the WT and the paralogue deletion was analyzed, as shown in Figure 6. The cell cycle resolved violin plots, seen in Figure 7 and in the Figures S6 and S7, visualize the distribution of mRNA counts across different cell cycle phases. The analysis of mRNA distributions showed significant differences in expression between WT and deletion strains for most genes, with a decrease in the frequency of cells with zero detected transcripts in deletion strains, suggesting compensatory upregulation.

In the *clb2*Δ strains, the proportion of cells without detectable *CLB1* mRNA decreased from 68% (WT) to 27%, with some cells in the mutant exhibiting transcriptional bursts with up to 31 detected *CLB1* spots, compared to WT. All phases showed a noticeable increase in *CLB1* expression, with more cells shifting toward higher transcript counts compared to WT. Especially the S and G2 phase exhibited a clear increase in *CLB1* expression, pointing to transcriptional compensation. In the *clb1*Δ strains, the proportion of cells without detectable *CLB2* mRNA decreased from 62% in the WT to 41% in the *clb1*Δ strains, with some cells in the mutant showing up to 23 detected *CLB2* spots. Notably, a greater number of spots were detected in the G2-budded phase of the mutant, suggesting increased transcriptional activity in this specific cell cycle stage.

Similarly, the *CLB3/CLB4* pair exhibited transcriptional compensation. The *CLB3* detection in the WT and *clb4*Δ mutant revealed that the proportion of cells without detectable *CLB3* mRNA (0 spots) was lower in the *clb4*Δ strain compared to the WT, consistent with compensatory upregulation. Similarly, the proportion of cells showing only one detectable *CLB3* spot was also reduced in the *clb4*Δ strain. Instead, a greater fraction of cells in the *clb4*Δ mutant exhibited two or more *CLB3* spots, with some cells showing up to 17 spots, and rare outliers reaching 23 spots. This suggests a redistribution of *CLB3* expression, with fewer cells displaying low expression levels (0–1 spots) and more cells exhibiting moderate to high expression levels (2+ spots). The upregulation of *CLB3* in the *clb4*Δ strain was observed across all cell cycle phases. The *CLB4* detection in the WT and *clb3*Δ deletion strains showed a trend consistent with compensatory upregulation, with fewer cells lacking detectable *CLB4* mRNA in the *clb3*Δ strain compared to WT. A slight increase in the fraction of cells with multiple *CLB4* spots was observed, with some cells showing up to 13 spots. However, unlike the *clb4*Δ mutant, the shift toward higher expression levels in the *clb3*Δ strain was less pronounced, and fewer cells exhibited high numbers of *CLB4* spots, suggesting weaker compensatory upregulation and limited transcriptional bursting. Additionally, no phase-specific upregulation was observed for *CLB4* in the *clb3*Δ strain.

In contrast, *CLB5/CLB6* showed little to no transcriptional compensation. No strong compensatory upregulation was observed in either deletion mutant. In the *clb6*Δ strain, the proportion of cells without detectable *CLB5* mRNA remained stable at around 60%, with no major shifts in expression across cell cycle phases. In the *clb5*Δ strain, the proportion of cells without detectable *CLB6* mRNA increased from 68% in WT to 76%, and the fraction of cells with moderate-to-high *CLB6* expression (2+ spots) was lower than in WT, dropping by more than half. Rather than showing signs of compensatory upregulation, *CLB6* expression was further reduced in the absence of *CLB5*. This pattern contrasts with other cases of transcriptional compensation, such as *CLB1/CLB2* and *CLN1/CLN2*, where deletion of one gene led to a clear increase in expression of the other. Instead, these results suggest that *CLB5* and *CLB6* do not engage in meaningful compensatory regulation at the transcriptional level.

Both *CLN1* and *CLN2* exhibited inherently bursty transcription in our data, characterized by sporadic but high-magnitude transcriptional events. This pattern was observed in both WT and deletion strains, with some cells exhibiting exceptionally high transcript counts. For *CLN1*, the proportion of cells without detectable *CLN1* mRNA decreased from 60% in WT to approximately 33% in the *cln2*Δ strain. The mutant also exhibited a greater fraction of cells with high transcript counts, indicating more frequent transcriptional bursts compared to WT. In the *cln2*Δ mutant, cells showed up to 44 detected *CLN1* spots, although rare outliers with even higher counts (up to 52 spots) were observed. Upregulation was seen across all phases, with a slightly stronger increase during late M phase, suggesting a phase-dependent compensatory response. However, in the *cln1*Δ strain, the proportion of cells without detectable *CLN2* mRNA did not decrease but instead slightly increased, from 58% in WT to 62% in the mutant, suggesting no strong, widespread compensatory upregulation at the population level. While rare transcriptional bursts reaching up to 59 detected spots were observed in individual cells, these appeared as isolated events rather than a general shift toward higher expression. Notably, the *p*-values of the Mann-Whitney U test indicate that *CLN2* has the highest *p*-value ($4.85\times 10^{-9}$) among the tested pairs, despite still being extremely small. Given the high number of analyzed cells, even such differences in *p*-values may suggest varying degrees of transcriptional divergence. While all tested gene pairs show highly significant differences, some exhibit even stronger shifts in expression patterns than others.

Unlike *CLN1* in *cln2*Δ, where a clear increase in high-expression cells was seen, the response of *CLN2* in *cln1*Δ suggests an asymmetrical compensation dynamic, where deletion of *CLN2* induces a stronger compensatory response in *CLN1* than vice versa. Additionally, no phase-specific regulation was observed for *CLN2* in the *cln1*Δ strain, reinforcing the idea that its compensatory potential might be more constrained or subject to additional regulatory mechanisms. Both *CLN1* and *CLN2* displayed bursty transcriptional behavior, with sporadic but pronounced increases in transcript counts. This suggests their regulation may involve stochastic transcriptional pulses, enhanced under compensatory conditions.

*SWI5* showed little to no evidence of genetic compensation upon deletion of *ACE2*, aside from a slight increase in transcription counts during the S phase in the mutant, which was minimal and does not indicate meaningful compensatory upregulation. However, we were unable to perform the corresponding experiments for the deletion of *SWI5*, preventing us from assessing whether *ACE2* could compensate in its absence due to the low number of detected mRNA molecules, making it difficult to distinguish from background noise.

Boxenplots in the Figures S8 and S9 summarize the central tendency of mRNA expression levels for the WT and the deletion strains. For *clb2*Δ strains, the mean mRNA count for *CLB1* increased from 0.8 in WT to 3.8 in deletion, while in *clb1*Δ strains, the mean mRNA count for *CLB2* increased from 0.7 in WT to 1.3 in deletion. Across some of the other paralogue pairs, a general pattern of compensatory upregulation was evident, with notable increases in mean transcript counts for one paralogue when its partner was deleted. This trend was particularly pronounced in pairs such as *CLB3/CLB4* and *CLN1/CLN2*, where the deletion of one gene led to a redistribution of expression levels, shifting towards higher transcript counts for the remaining paralogue. Table 3 presents the summary of the results.

### 3.6. Phenotype of Deletion Strains

We used the cell segmentation from Cellpose 3.0 also as a measure of cell size and for comparison of the deletion strains with the WT strain. Most of the B-Cvclin deletions lead to bigger cells, while *clb1*Δ and *clb3*Δ show smaller cells (Figures S10–S12). Deletions of *cln1*Δ and *cln2*Δ show less significant size effects, with only a moderate increase for *cln2*Δ, while *swi5*Δ and *ace2*Δ have no influence on cell size. Deletion of *she2*Δ leads to a slight increase in cell size. We also measured doubling time by absorbance recording of the strains used in 96 well plates in YPD medium. Recording was performed every 30 min and results were fitted with an exponential function. From the exponent giving the generation time, doubling time was calculated by dividing ln2 with generation time. Mean doubling times are shown in Supplementary Figure S13. Besides the *sic1*Δ deletion, the other strains used show no significant growth delay.

## 4. Discussion

In this study, we have investigated several thousand single cells per experiment in order to shed light on the differential expression of a set of paralogous yeast cell cycle genes, which are often treated like being the same. To this end, we used single-molecule inexpensive fluorescence in situ hybridization (smiFISH) and counted always groups of three types of single mRNA molecules per cell. We found differences in the expression of the paralogues, but we also found different characteristics between the pairs. While *CLB3*/*CLB4* exhibits a difference in expression, *CLB1*/*CLB2* shows a difference in their localization (enrichment of *CLB2* in the bud), *CLN1* reacts with an up-regulation, differently to the deletion of its counterpart than *CLN2*.

While our study observed these effects, not all of them can be explained by our findings and would require alternative experimental approaches. Here, we can conclude the following: While *CLN1* and *CLN2* are very similar and share many regulatory mechanisms, there are some subtle differences in their expression regulation, with higher number of observations for *CLN2*. Their similarities comprise that (i) Both *CLN1* and *CLN2* are activated by the transcription factors SBF and MBF, which bind to SCB and MCB elements in their promoters, respectively. This shared regulation ensures their coordinated expression during late G1, (ii) both *CLN1* and *CLN2* are positively regulated by Cln3p, which contributes to their transcriptional activation. (iii) Both *CLN1* and *CLN2* are negatively regulated by G2 cyclins (Clb1p, Clb2p, Clb3p, Clb4p), ensuring their repression during G2 phase [27], and eventually (iv) both *CLN1* and *CLN2* are targets of Far1p, which inhibits their activity in response to pheromone signaling [28]. Their differences are first quantitative differences: While both *CLN1* and *CLN2* are expressed in late G1 [25], we found subtle quantitative differences in their expression levels. Studies suggest that *CLN1* might be more sensitive to changes in carbon sources and play a more significant role in adapting cell size to nutrient availability [29]. Although both Cln1p and Cln2p proteins are distributed between the nucleus and cytoplasm, Cln1p shows a stronger nuclear accumulation compared to Cln2p. This difference in localization might lead to subtle variations in their interactions with other proteins and their downstream effects. While they have overlapping functions, they might have slightly different specific roles in cell cycle progression. For example, *CLN1* has been implicated in pseudohyphal development, a filamentous growth form in yeast, while *CLN2* might have a more prominent role in other aspects of cell cycle control [30]. In our study we see a slight expression peak for *CLN1* and *CLN2* in S phase, but have to be aware that the discrimination of late G1 from S phase is not perfect. The oscillatory behavior in our study is probably blurred by the inaccuracy of phase assignment. We see a clear overrepresentation of G1 phase and an underrepresentation of S phase in all experiments. S phase assignment is complicated by the fact, that small buds are often not segmented by Cellpose 3.0.

*CLB5* and *CLB6* are a pair of B-type cyclins crucial for the initiation of DNA replication during the S phase of the cell cycle [31]. They share many similarities in their regulation, but also exhibit some key differences. Among the similarities are that both *CLB5* and *CLB6* are primarily activated by the transcription factor complex MBF (Mbp1p/Swi6p). MBF binds to MCB elements in the promoters of both genes, driving their expression during late G1 phase, just before the onset of S phase. Their coordinated expression ensures the availability of these cyclins when needed for DNA replication. Both Clb5p and Clb6p, when bound to Cdc28p (a cyclin-dependent kinase), form active complexes that are essential for initiating DNA replication. They contribute to the phosphorylation and activation of proteins involved in the assembly of the pre-replicative complex (pre-RC) at replication origins. Their differences are first slight quantitative differences: While both cyclins are expressed in late G1, there might be subtle quantitative differences in their expression levels. Studies suggest that Clb5p might be more abundant than Clb6p, implying potential differences in their contributions to DNA replication [32]. While MBF is the primary activator, other factors might influence the expression of *CLB5* and *CLB6* to varying degrees. For example, some evidence suggests that Cln1p and Cln2p, the G1 cyclins, might also play a role in the activation of *CLB5* and *CLB6* transcription, though the precise mechanisms are not fully understood [33]. While they both Clb5p and Clb6p contribute to DNA replication initiation, they might have slightly different specific roles in the process. For instance, some studies suggest that Clb5p might be more important for the initiation of replication at specific origins, while Clb6p might play a more general role in ensuring the overall progression of S phase [34,35]. In our study we found lower mRNA numbers for *CLB5* and *CLB6* in respect to other B-cyclins and no strong oscillatory behavior. Deletion strains of *clb5*Δ and *clb6*Δ develop bigger cells and *clb5*Δ has a small growth delay in respect to WT (see Supplementary Materials). *CLB5* and *CLB6* show no evidence of transcriptional compensation upon deletion of their respective paralogue.

B-type cyclins *CLB3* and *CLB4* play important roles in the G2/M transition of the cell cycle. They are mainly expressed during S phase and G2, with their mRNA and protein levels peaking in G2, just before the onset of mitosis. This coordinated expression ensures their availability for the G2/M transition [25]. Both Clb3p and Clb4p, when bound to Cdc28p, form active complexes that are essential for promoting the G2/M transition. They contribute to the phosphorylation and activation of proteins involved in mitotic spindle assembly and other processes required for cell division. Unfortunately, the precise upstream regulators of *CLB3* and *CLB4* are not as well-defined as those for *CLB5/CLB6*, but they are likely influenced by upstream activators and repressors that coordinate their expression with the cell cycle progression. For the protein level, studies suggest quantitative differences, i.e., that Clb3p might be more abundant than Clb4p, implying potential differences in their contributions to the G2/M transition [36]. This difference in abundance is confirmed by our expression data, we also see lower numbers for *CLB4* mRNA than for *CLB3*. Although both contribute to the G2/M transition, *CLB3* and *CLB4* might have slightly different specific roles in the process. For instance, some studies suggest that Clb3p might be more important for certain aspects of spindle assembly, while Clb4p might play a more prominent role in other mitotic events [37]. Genetic studies have revealed some differences in the interactions of *CLB3* and *CLB4* with other genes. These differences might reflect subtle variations in their functions and their contributions to different cellular pathways [33].

*CLB1* and *CLB2* are another–final–pair of B-type cyclins crucial for the G2/M transition, which is the point in the cell cycle where the cell prepares to divide. Both *CLB1* and CLB2 are known to be expressed during the S and G2 phases of the cell cycle, with their levels peaking in G2, right before the cell enters mitosis (M phase) [25]. This coordinated timing ensures that these proteins are available when needed for cell division. Both Clb1p and Clb2p, when they bind to Cdc28p (a cyclin-dependent kinase), form active complexes that are essential for promoting the G2/M transition [38]. They help to activate proteins involved in mitotic spindle assembly and other processes necessary for cell division. Both Clb1 and Clb2 proteins are degraded at the end of mitosis. This degradation is crucial for the proper progression of the cell cycle and prevents premature entry into the next cell cycle. While both cyclins are expressed in S and G2, there might be subtle differences in the amount of each protein produced. Studies suggest that Clb2p is generally more abundant than Clb1p, implying that Clb2p might play a more dominant role in mitosis. Although they both contribute to the G2/M transition, *CLB1* and *CLB2* might have slightly different specific roles in the process. For instance, some research indicates that Clb1p might be more important for meiosis (cell division for sexual reproduction), while Clb2p is more crucial for mitosis (cell division for growth and repair) [39]. While the exact upstream regulators of *CLB1* and *CLB2* are still being studied, it’s likely that they are influenced by other factors that coordinate their expression with the cell cycle. For example, some studies suggest that the RNA-binding protein Puf5p and the HMGB protein Ixr1p might play a role in regulating *CLB1* expression [40]. Our smiFISH gene expression analysis revealed the clearest oscillation for *CLB1*/*CLB2* among the analyzed paralogues. Both peak in G2 phase with *CLB1*/having the higher total abundance, while *CLB2* is enriched in the bud. For *CLB2* a She2p-She3p dependent mRNA transport was shown [26], why we analyzed the pair as well in the *she2*Δ background, were active transport should be abolished. We see for both *CLB1* and *CLB2* compensation for the paralogue and we see also for both up-regulation in the *she2*Δ background. Interestingly the up-regulation is mainly visible in the mother cells, while the mRNA numbers in the bud stay unchanged, why we speculate, that expression is turned on until a level, where enough mRNA enter the bud by simple diffusion or in other words, until the bud is satisfied. Surprisingly, this is the case for both species. Whether this means, that there is also *CLB1* transport to the bud, we can not rule out.

*ACE2* and *SWI5* are two homologous transcription factors that play crucial roles in the late stages of mitosis and early G1 phase, particularly in daughter cell development [25]. Both Ace2p and Swi5p possess highly conserved zinc finger DNA-binding domains, enabling them to recognize and bind to the same DNA sequences in the promoters of their target genes. Both proteins are active in late mitosis and early G1, specifically in daughter cells [41]. Their activity is tightly regulated by their nuclear localization, which is controlled by phosphorylation events. And both Ace2p and Swi5p are involved in the transcriptional activation of genes required for daughter cell separation (septum degradation) and bud site selection. However, they also show important differences. Despite sharing similar DNA-binding domains, Ace2p and Swi5p regulate distinct sets of genes [42]. Ace2p primarily activates genes involved in septum degradation (e.g., CTS1, SCW11) and bud site selection (e.g., BUD9), while Swi5p regulates genes with different functions [43]. Ace2p, but not Swi5p, represses the transcription of *CLN3*, a G1 cyclin [44]. This repression contributes to the longer G1 phase observed in daughter cells compared to mother cells, allowing them to grow sufficiently before budding. While both proteins are nuclear during their active phase, their nuclear localization is differentially regulated. Ace2p’s nuclear entry and exit are controlled by phosphorylation events mediated by Cbk1p, which specifically targets daughter cells. Swi5p’s localization might be regulated by different mechanisms [45]. Although both contribute to cell separation, they might have slightly different roles. Ace2p is essential for the degradation of the septum between mother and daughter cells, while Swi5p might be involved in other aspects of cell separation or daughter cell development. For *ACE2* and *SWI5* our expression data differ to previously reported results. Rather than the expected expression in late mitosis/G1, we observe peak expression in G2. Whether this results from a time delay between transcription and translation remains uncertain. Interestingly, we see no compensation of *SWI5* for *ace2*Δ.

In addition to gene expression changes, we also observed differences in cell size in certain deletion mutants. Normalized cell area distributions (Figures S10–S12) revealed that *clb1*Δ and *clb3*Δ mutants exhibited smaller cell sizes compared to WT, whereas *clb2*Δ and *clb4*Δ mutants showed size distributions similar to WT. Additionally, *clb6*Δ cells appeared slightly larger, a trend also visible in microscopy images. The remaining deletion strains displayed no notable deviations in cell size.

Other publications focusing on FISH data are analyzing next to mature mRNA also transcription sites (e.g., [46]). Our targets are very low expressed and we see transcription sites not regularly, even in bursts they are mostly not seen, why we did not include transcription site analysis.

Compared to other studies that synchronized yeast cell cycle by adding α-factor (e.g., [47]), we used asynchronous cell populations in order to least perturb the expression and cell growth during different cell cycle phases.

Taken together, the oscillation behavior of most of our targets corresponds to literature. The mean values per phase are potentially slightly too low in the peaks and too high in G1 due to imperfect phase assignment. The reason is that in our cell cycle phase assignment model the number of G1 phase cells is always overestimated and the number of cells in S phase is underestimated, because (i) the small bud is sometimes not seen and (ii) also the spindle pole body, which separates budding cells between S phase (1 SPB) and G2 (2 SPB), is in quite a number of cases not visible, which also may lead to wrong assignment.

Our data gives also rise to reason about whether expression of the considered genes is more steady or more burst-like, though without mechanistic explanation. The low number of peaks of both *CLB3* and *CLB4* together with their low number of cells without any peak would be more supportive of a continuous basal expression, while high peaks and many cells without any peak seem to point to more burst-like expression of *CLN1* and *CLN2*. Here, we should also take into account half-life times, which have been reported as 5.03 min for *SIC1*, 5.98 min for *CLN1*, 7.43 min for *CLN2*, 7.45 min for *CLB1*, 4.95 min for *CLB3*, 3.76 min for *CLB4*, 6.12 min for *CLB5*, 4.84 min for *CLB6*, 6.6 min for *CDC28*, 7.49 for *SWI5* and finally 5.74 min for *ACE2* [48]. This means, that mRNA molecules of *CLN2* and *CLB1* would have a higher probability of being detected once expressed than mRNA molecules of *CLB3* or *CLB4*, which are faster degraded.

## Figures and Tables

**Table 1:** Gene Information Table. The table lists gene names, NCBI accession numbers, percentage identity of translated protein sequences determined using BLASTp, percentage identity of DNA sequences determined using BLASTn, chromosomal locations, and gene sizes in base pairs (bp).

Gene	Accession Number	Percentage Identity Protein (blastp)	Percentage Identity DNA (blastn)	Chromosome	Gene Size (bp)
*CLB1*	YGR108W	74.47%	72.75%	VII	1416
*CLB2*	YPR119W			XVI	1476
*CLB3*	YDL155W	49.40%	66.76%	IV	1284
*CLB4*	YLR210W			XII	1383
*CLB5*	YPR120C	47.70%	64.67%	XVI	1308
*CLB6*	YGR109C			VII	1143
*CLN1*	YMR199W	57.66%	68.06%	XIII	1641
*CLN2*	YPL256C			XVI	1638
*SWI5*	YDR146C	46.84%	70.12%	IV	2130
*ACE2*	YLR131C			XII	2313

**Table 2:** Pearson correlation coefficients for paralogues gene pairs.

Paralogue Pair	Pearson Correlation Value
*CLB1/CLB2*	0.51
*CLB3/CLB4*	0.26
*CLB5/CLB6*	0.49
*CLN1/CLN2*	0.68
*SWI5/ACE2*	0.58

**Table 3:** Summary of observations.

	Observations		
Paralogue Pair	Positive Correlation	Specific Mother/Bud Localization	Genetic Compensation
*CLB1/CLB2*	Moderately	Yes	Yes
*CLB3/CLB4*	Weakly	No	Yes
*CLB5/CLB6*	Moderately	No	No
*CLN1/CLN2*	Strongly	No	*CLN1* yes, *CLN2* no
*SWI5/ACE2*	Moderately	No	No

## Data Availability

All data is represented in the Supplementary Materials. We can make all data available upon request. The CNN algorithm is available at https://ford.biologie.hu-berlin.de/frenner/yeastparalogues25 (accessed on 13 February 2025).

## Supplementary Materials

The following supporting information can be downloaded at: https://www.mdpi.com/article/10.3390/cells14060412/s1, Figure S1. Left: Teflon rack for coverslip cleaning and activation. Right: Silicon chamber attached to activated coverslip; Figure S2. Boxenplots for spots detected and scatter plots with replicate information. For every pair, each colour represent one experiment; Figure S3. Mean value of spots observed in cells with 0, 1 and 2 SPB. Cells with 0 SPB are mainly S Buds and G2 Buds. 1 SPB cells correspond mostly to G1, S and M phases and 2 SPB only account G2 phase; Figure S4. Distribution and mean number of spots per cell for the different cell-cycle phases in the WT CLB paralogues; Figure S5. Distribution and mean number of spots per cell for the different cell-cycle phases in the WT CLN paralogues and for SIC, SWI5 and ACE2; Figure S6. Half-violin plots showing transcript count distributions across the cell cycle for WT (left) and deletion strains (right); Figure S7. Half-violin plots showing transcript count distributions across the cell cycle for WT (left) and deletion strains (right); Figure S8. Boxenplots and scatterplots with number of spots observed for each gene in WT strain, on the left, and in the deletion of its paralogue, on the right; Figure S9. Boxenplots and scatterplots with number of spots observed for each gene in WT strain, on the left, and in the deletion of its paralogue, on the right; Figure S10. Normalized segmented cell area distributions of clbΔs versus WT cells; Figure S11. Normalized segmented cell area distributions of clnΔs versus WT cells and ace2Δ and swi5Δ versus WT cells; Figure S12. Normalized segmented cell area distributions of she2Δ versus WT cells; Figure S13. Doubling times of BY4741 (WT) and several deletion mutants (D) in YPD batch cultures; Table S1. Pearson correlation coefficients for each replicate. We used four replicates for every pair, except for CLB5/CLB6, where we performed three experiments; Table S2. Pearson correlation coefficients for CLB1, CLB2, CLB3, CLB4, CLB5, CLB6, SWI5 and ACE2 with SIC1; Supplementary Methods: Spc42-mTurquoise tagging and Coverslip activation with APTES [18,20].

## Abbreviations

The following abbreviations are used in this manuscript:

CNN	Convolutional neural network
mRNA	Messenger RNA
smiFISH	Single molecule inexpensive fluorescent in situ hybridization
WGD	Whole genome duplication
WT	Wildtype
