# Supplementary material for: Expression Dynamics and Genetic Compensation of Cell Cycle Paralogues in Saccharomyces cerevisiae
Source: Cells. 2025 Mar 11;14(6):412. doi: 10.3390/cells14060412 (PMC11941160; doi:10.3390/cells14060412)
Supplement: Supplementary file 1 [file cells-14-00412-s001.zip › cells-3503070-supplementary.pdf]

Supplementary Material

Expression Dynamics and Genetic Compensation of Cell Cycle Paralogues in *Saccharomyces cerevisiae*

February 2025

Gabriele Schreiber, Facundo Rueda, Florian Renner, Asya Polat, Philipp Lorenz, and Edda Klipp

Contents

Supplementary Figure S1 — 2
Supplementary Figure S2 — 3
Supplementary Table S1 — 4
Supplementary Table S2 — 4
Supplementary Figure S3 — 5
Supplementary Figure S4 — 6
Supplementary Figure S5 — 7
Supplementary Figure S6 — 8
Supplementary Figure S7 — 9
Supplementary Figure S8 — 10
Supplementary Figure S9 — 11
Supplementary Figure S10 — 12
Supplementary Figure S11 — 13
Supplementary Figure S12 — 13
Supplementary Figure S13 — 14
Supplementary Methods — 15

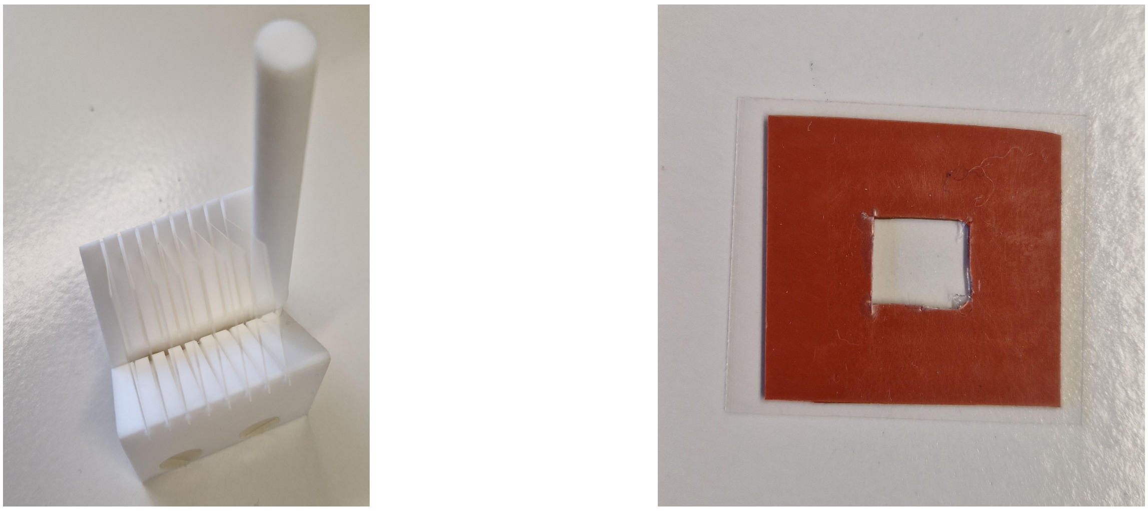

Supplementary Figure S1. Left: Teflon rack for coverslip cleaning and activation. Right: Silicon chamber attached to activated coverslip.

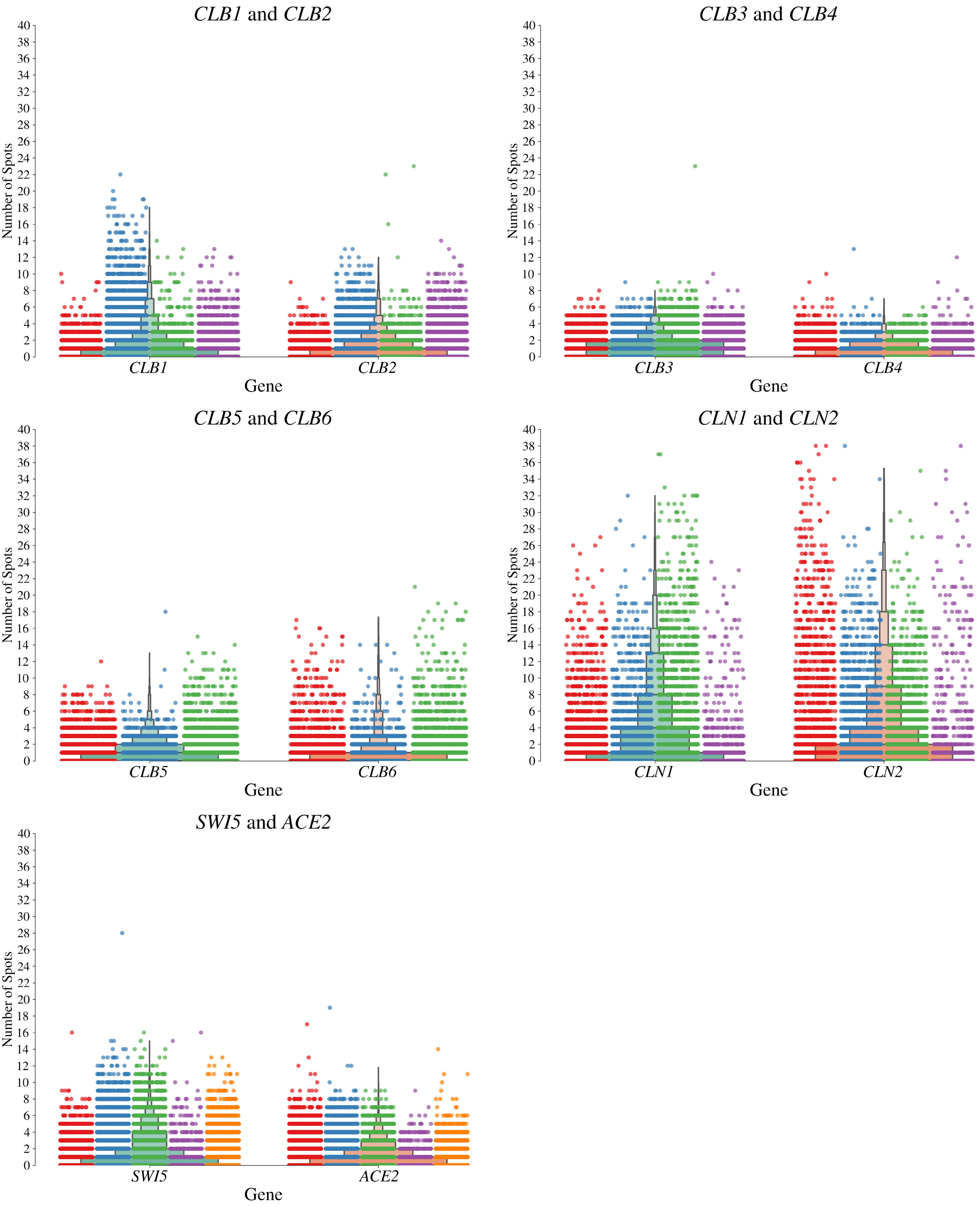

Supplementary Figure S2. Boxenplots for spots detected and scatter plots with replicate information. For every pair, each colour represent one experiment.

Paralogue pair	Replicate 1	Replicate 2	Replicate 3	Replicate 4
CLB1/CLB2	0.27	0.56	0.19	0.3
CLB3/CLB4	0.24	0.26	0.28	0.16
CLB5/CLB6	0.5	0.17	0.57	-
CLN1/CLN2	0.76	0.72	0.73	0.79
SWI5/ACE2	0.51	0.56	0.65	0.51

Supplementary Table S1. Pearson correlation coefficients for each replicate. We used four replicates for every pair, except for *CLB5/CLB6,* where we performed three experiments.

Paralogue pair	Replicate 1	Replicate 2	Replicate 3	Replicate 4
CLB1/SIC1	0.02	-0.04	-0.07	-0.05
CLB2/SIC1	0.02	-0.09	0.19	-0.08
CLB3/SIC1	-0.06	0.09	-	-
CLB4/SIC1	-0.01	0.06	-	-
CLB5/SIC1	0.12	0.15	-	-
CLB6/SIC1	-0.05	-0.01	-	-
SWI5/SIC1	-0.01	-0.04	-0.07	-0.03
ACE2/SIC1	-0.03	0.01	-0.05	0

Supplementary Table S2. Pearson correlation coefficients for *CLB1, CLB2, CLB3, CLB4, CLB5, CLB6, SWI5* and *ACE2* with *SIC1*.

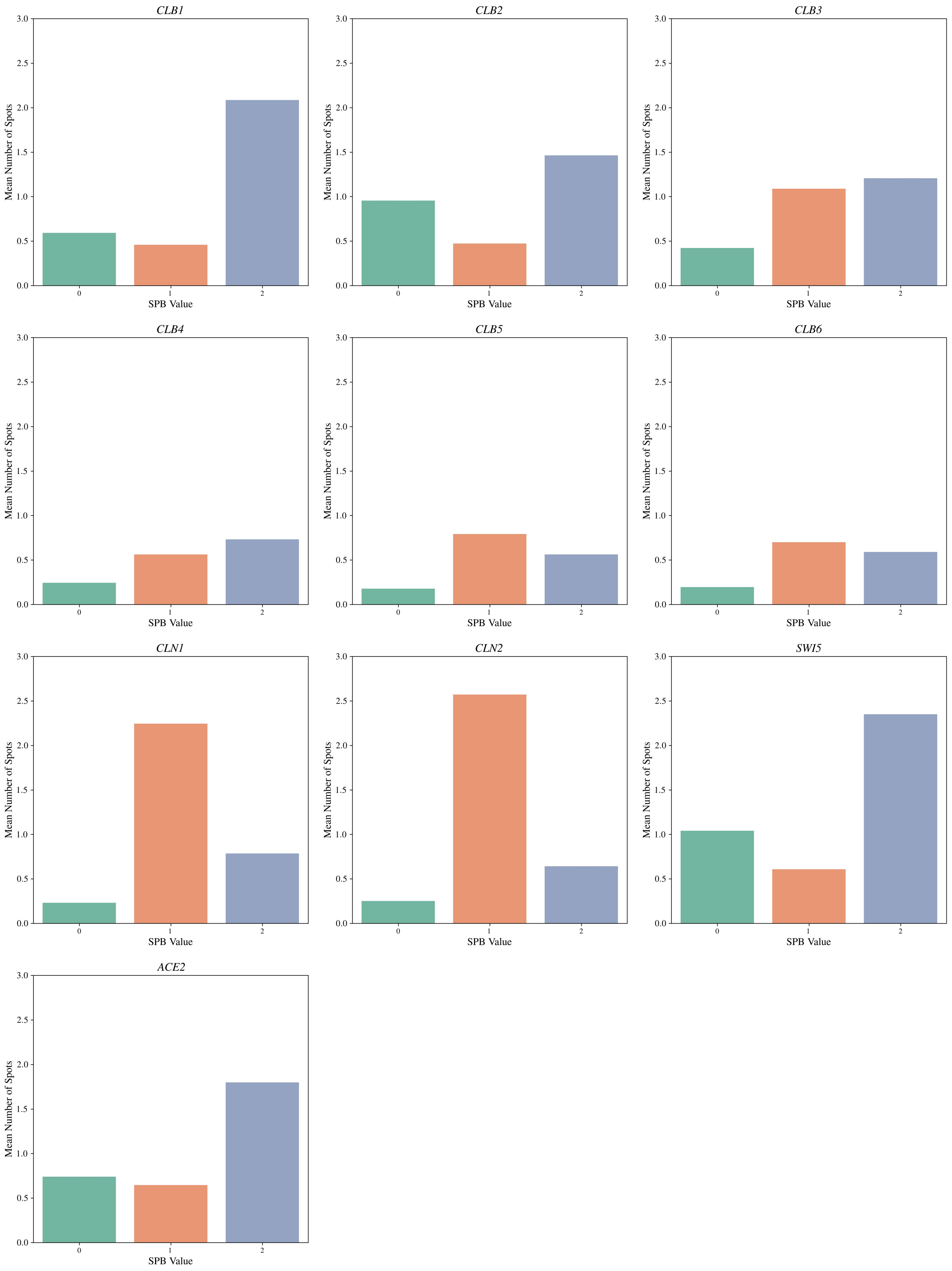

Supplementary Figure S3. Mean value of spots observed in cells with 0, 1 and 2 SPB. Cells with 0 SPB are mainly S Buds and G2 Buds. 1 SPB cells correspond mostly to G1, S and M phases and 2 SPB only account G2 phase.

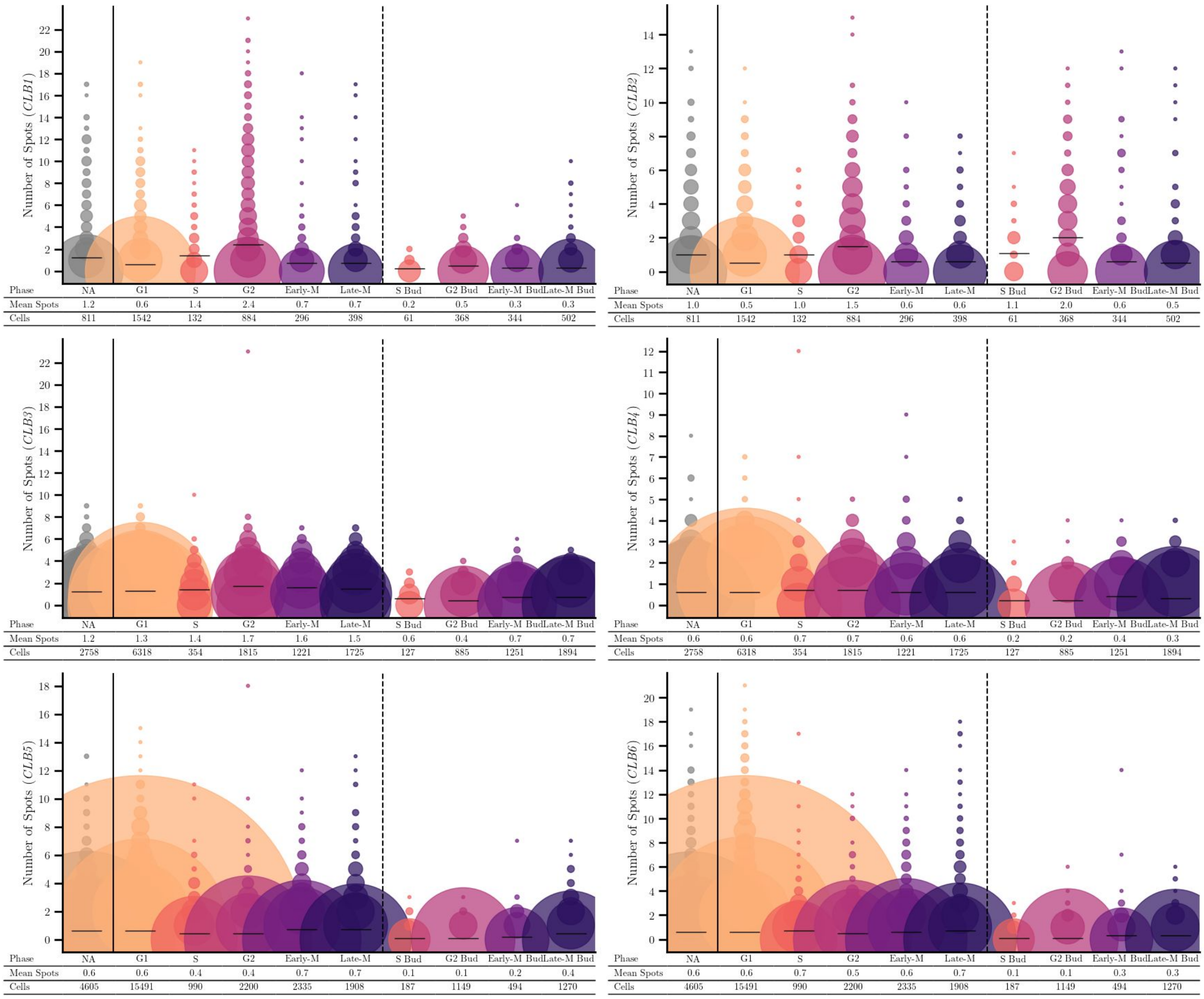

Supplementary Figure S4. Distribution and mean number of spots per cell for the different cell-cycle phases in the WT *CLB* paralogues.

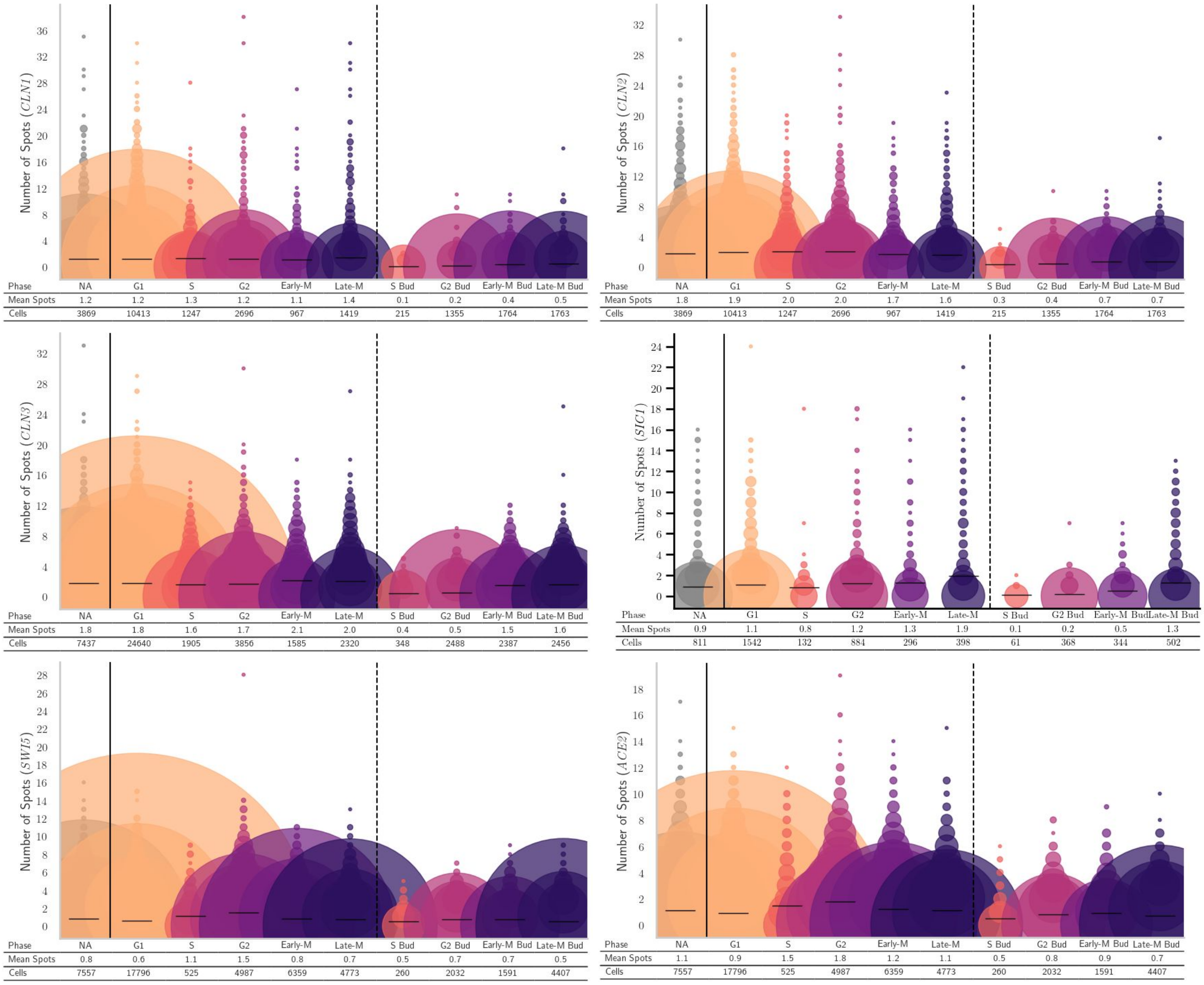

Supplementary Figure S5. Distribution and mean number of spots per cell for the different cell-cycle phases in the WT *CLN* paralogues and for *SIC*, *SWI5* and *ACE2*.

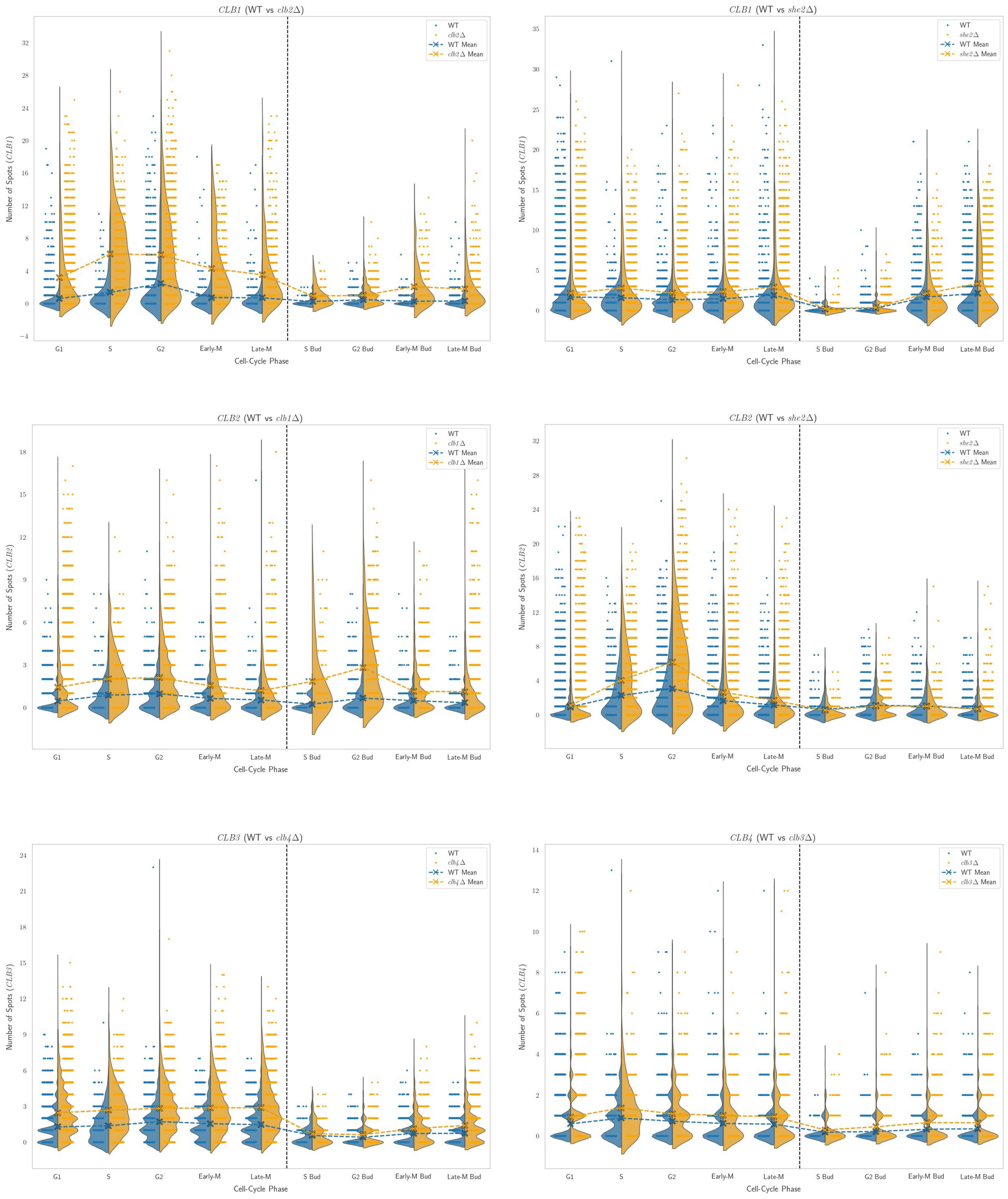

Supplementary Figure S6. Half-violin plots showing transcript count distributions across the cell cycle for WT (left) and deletion strains (right).

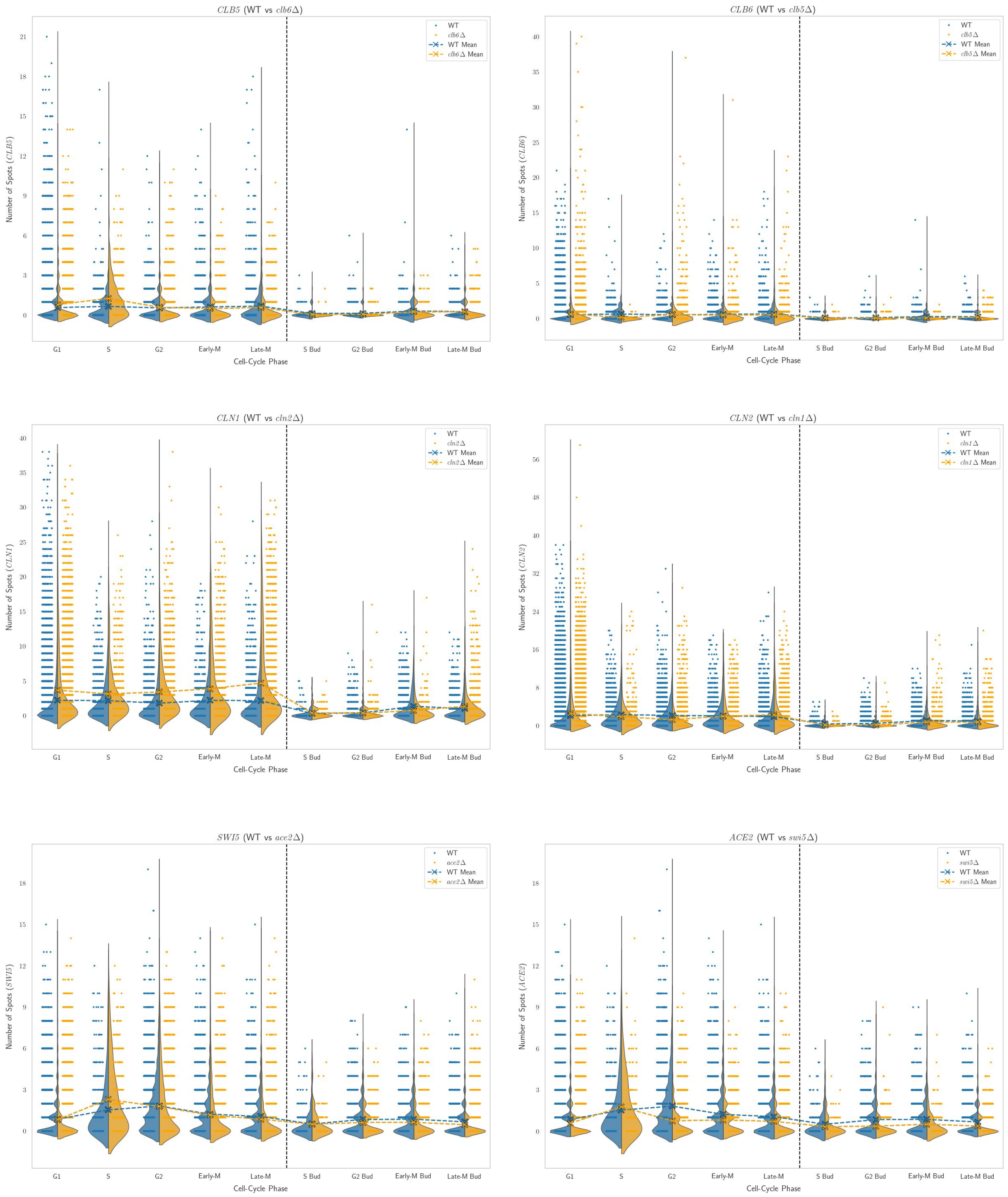

Supplementary Figure S7. Half-violin plots showing transcript count distributions across the cell cycle for WT (left) and deletion strains (right).

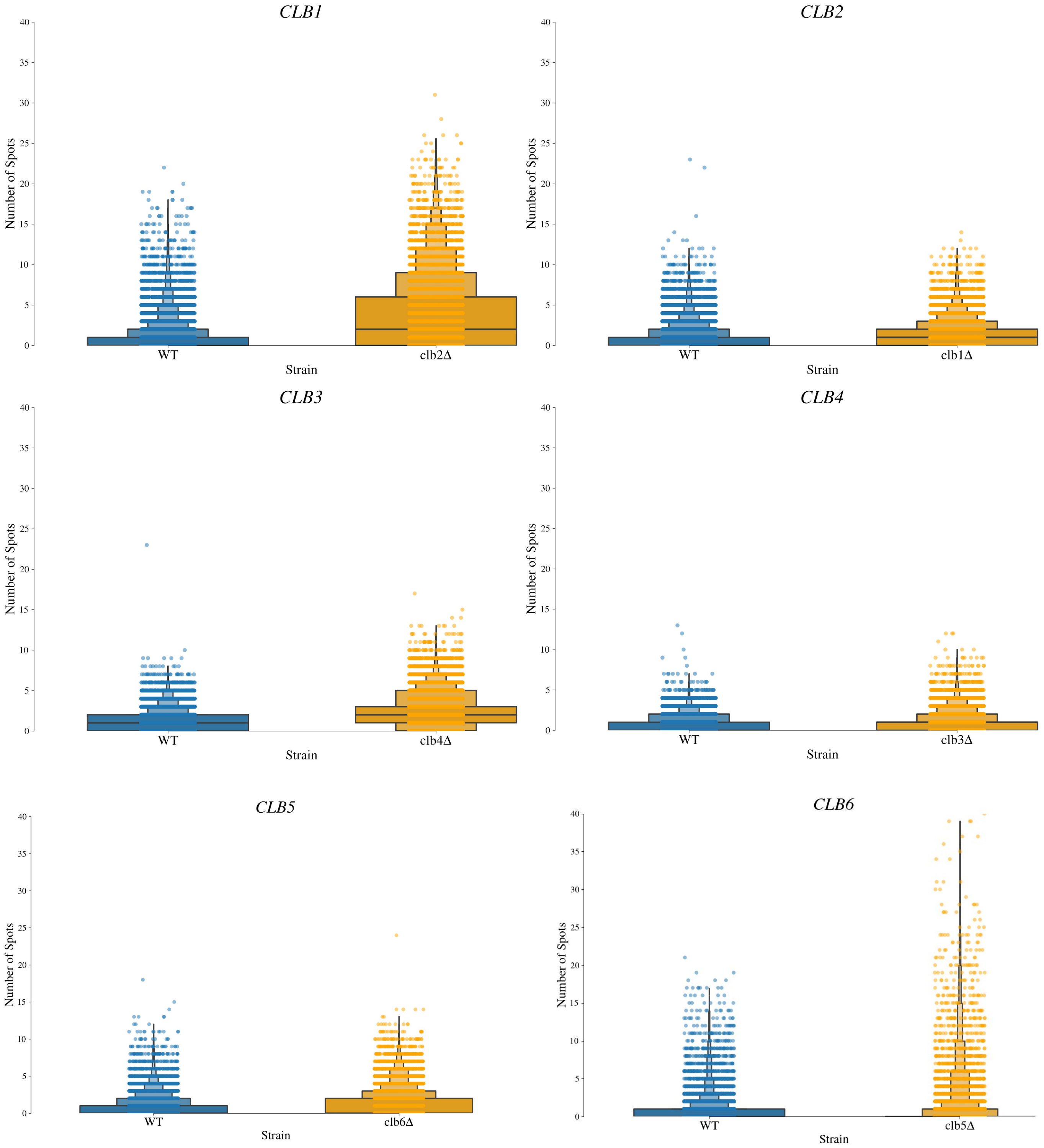

Supplementary Figure S8. Boxenplots and scatterplots with number of spots observed for each gene in WT strain, on the left, and in the deletion of its paralogue, on the right.

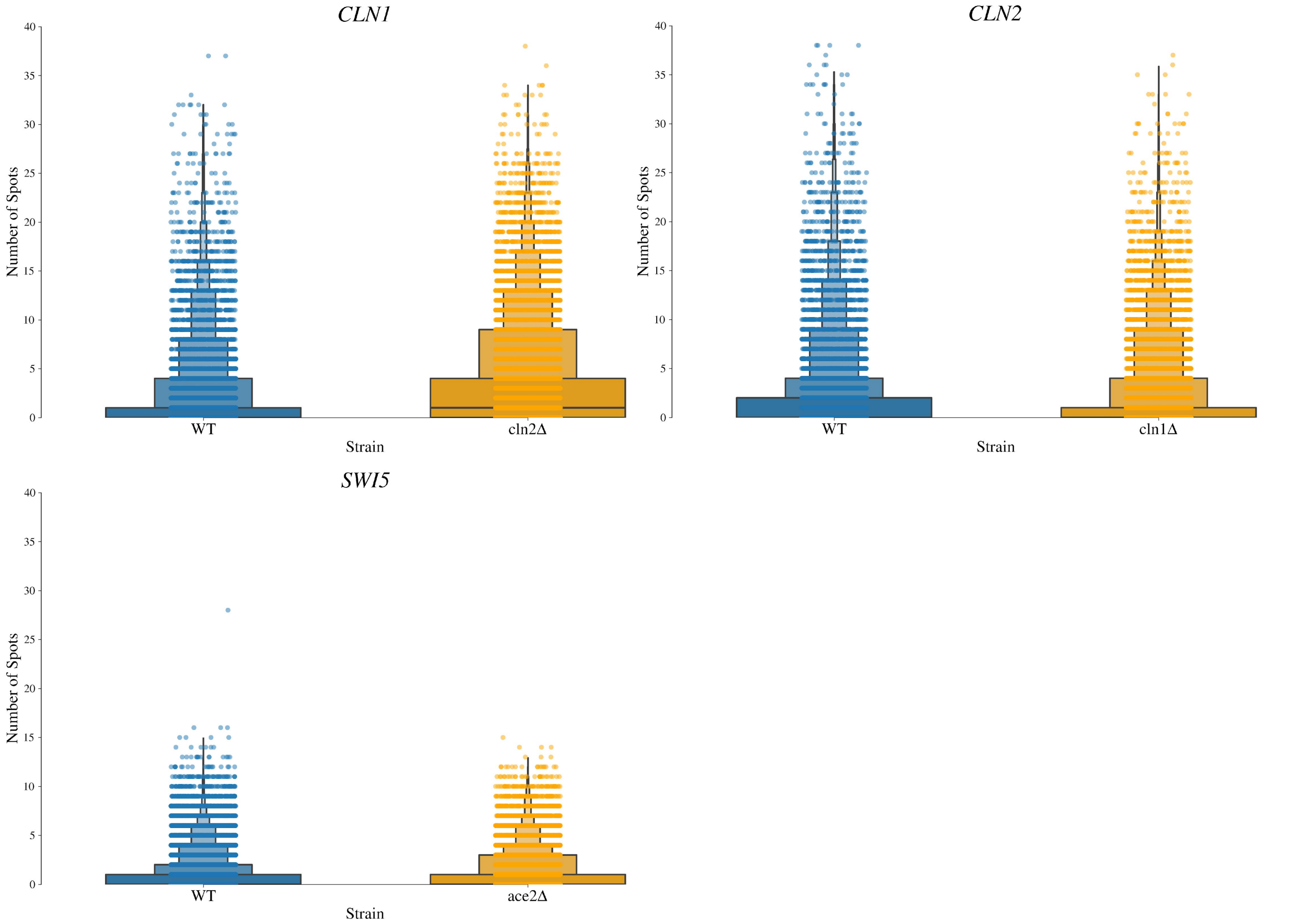

Supplementary Figure S9. Boxenplots and scatterplots with number of spots observed for each gene in WT strain, on the left, and in the deletion of its paralogue, on the right.

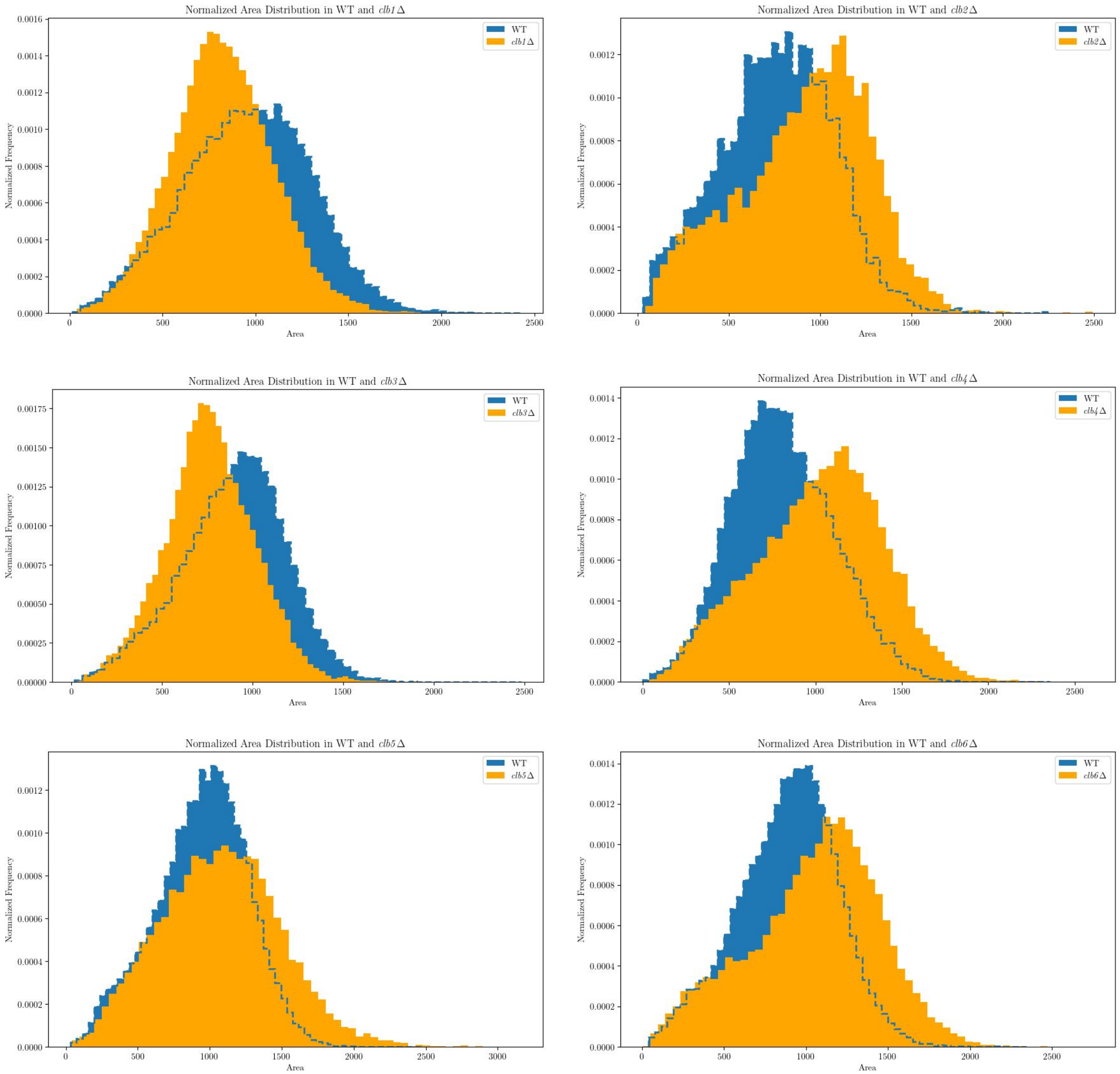

Supplementary Figure S10. Normalized segmented cell area distributions of clbΔs versus WT cells.

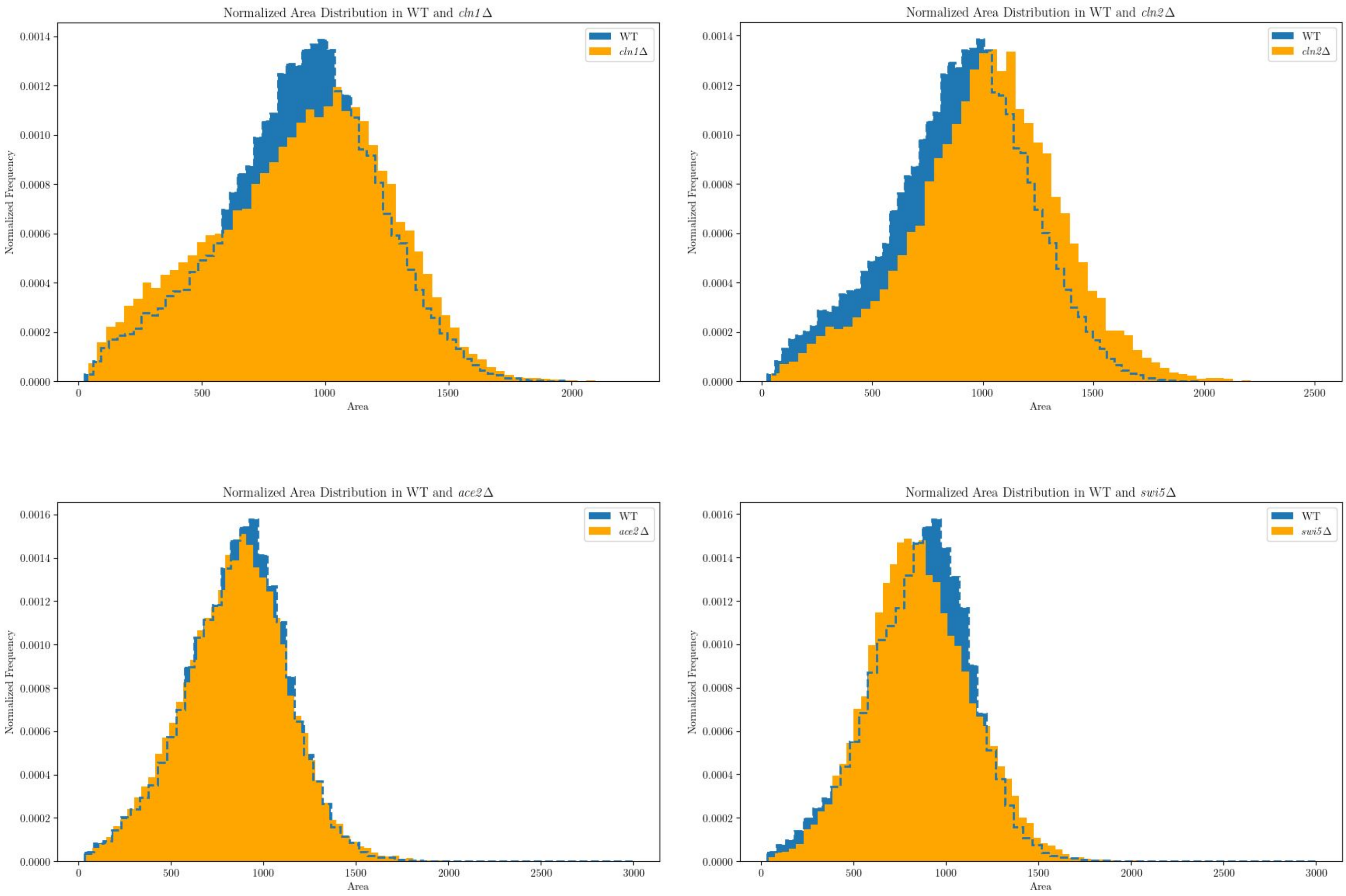

Supplementary Figure S11. Normalized segmented cell area distributions of clnΔs versus WT cells and ace2Δ and swi5Δ versus WT cells.

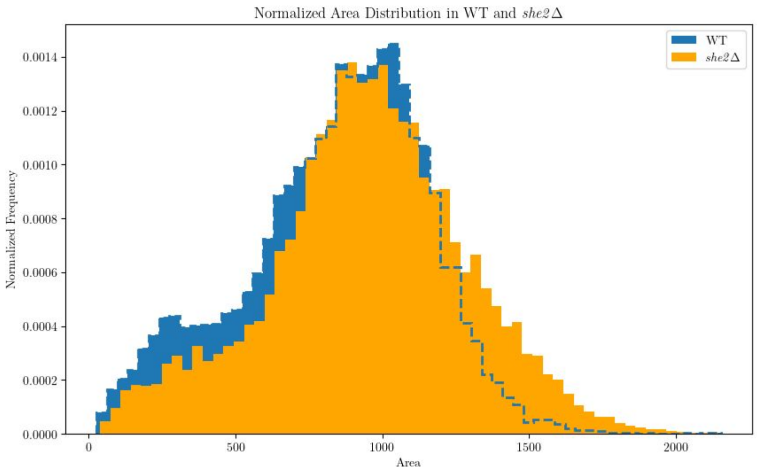

Supplementary Figure S12. Normalized segmented cell area distributions of she2Δ versus WT cells.

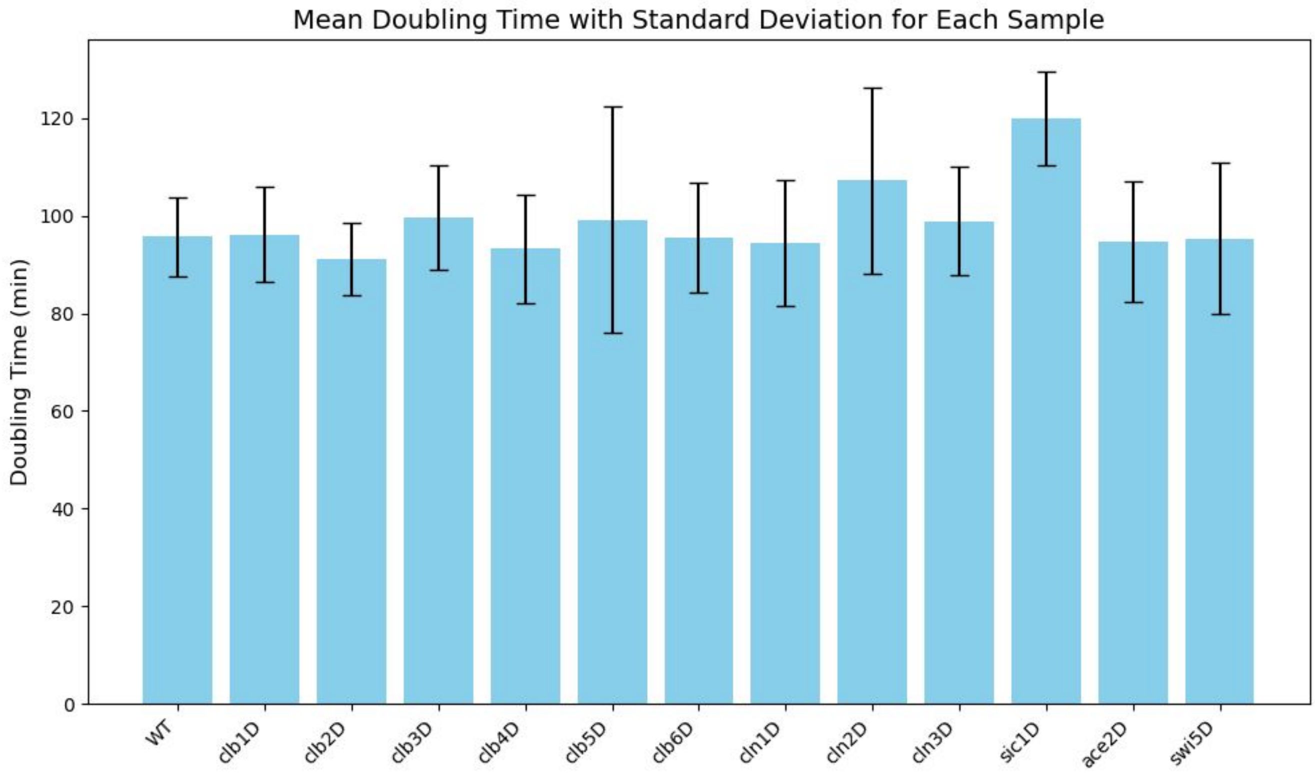

Supplementary Figure S13. Doubling times of BY4741 (WT) and several deletion mutants (D) in YPD batch cultures

Supplementary Methods

Spc42-mTurquoise tagging

We used pUG72 a vector designed for deletions with klUra3 as selection marker flanked by two loxP sites. We cloned mTurquoise (a GFP analogon) inside PstI restriction site with a 10 amino acid alternating glycine/alanine linker at the N-terminus of mTurquoise. For homologous recombination inside *SPC42* location, we PCR amplified the full 2387bp cassette containing mTurquoise and the *klUra3* gene with the primer pair:
5'-AAATAATATGTCAGAAACATTCGCAACTCCCACTCCCAATAATCGAGGAGCAGGTG CTGGTGC-3'/
5'-AACGCTTTAAGAATGCGCCATACTCCTTAACTGCTTTTTAAATCACCTAATAACTTCG TATAGC-3'.
The PCR Product was transformed into yeast BY4741 stains with the protocol developed by Daniel Gietz [18]. Positive clones were selected on SD medium lacking uracil, and confirmed by microscopy.

Coverslip activation with APTES

For coverslip activation we use a modified protocol from Lubeck et al. [20]. We use self made holders out of teflon (Figure S11). Coverslips (VWR 631-0144) were mounted into the holder and washed in 1% Alconox (Sigma-Aldrich Z273228) under sonication. Temperature in the sonicator is set to 45◦C and first sonification is performed for 5 min at 100% power with degas function. Afterwards sonification at 100% was prolonged for 25 min without degas function. After washing in detergent, coverslips were rinsed twice for 2 min in ultra pure water. The water was changed again and coverslips were sonicated for 5 min at 100% and 45◦C. Coverslips were left to dry. Dry coverslips were cleaned with a plasmacleaner for 5 min at median power. Afterwards the teflon holders with the clean coverslips were submerged for to 2 min in a 2% 3-Aminopropyltriethoxysilane (APTES) (Sigma 440140) solution in chloroform and afterwards rinsed with ultra pure water before air drying.
